# Phosphodiesterase type 5 inhibitors enhance chemotherapy in preclinical models of esophageal adenocarcinoma by targeting cancer-associated fibroblasts

**DOI:** 10.1016/j.xcrm.2022.100541

**Published:** 2022-06-21

**Authors:** Benjamin P. Sharpe, Annette Hayden, Antigoni Manousopoulou, Andrew Cowie, Robert C. Walker, Jack Harrington, Fereshteh Izadi, Stella P. Breininger, Jane Gibson, Oliver Pickering, Eleanor Jaynes, Ewan Kyle, John H. Saunders, Simon L. Parsons, Alison A. Ritchie, Philip A. Clarke, Pamela Collier, Nigel P. Mongan, David O. Bates, Kiren Yacqub-Usman, Spiros D. Garbis, Zoë Walters, Matthew Rose-Zerilli, Anna M. Grabowska, Timothy J. Underwood

**Affiliations:** 1 School of Cancer Sciences, Faculty of Medicine, Room CS B2, MP824, Somers Cancer Research Building, University Hospital Southampton, Tremona Road, Southampton SO16 6YD, UK; 2 Proteas Bioanalytics, Torrance, CA 90502, USA; 3 Centre for NanoHealth, Swansea University Medical School, Singleton Campus, Swansea SA2 8PP, UK; 4 University Hospital Southampton NHS Foundation Trust, Southampton SO16 6YD, UK; 5 *Ex Vivo* Cancer Pharmacology Centre of Excellence, School of Medicine, Biodiscovery Institute, University of Nottingham, Nottingham NG7 2RD, UK; 6 Salford Royal NHS Foundation Trust, Salford M6 8HD, UK; 7 Nottingham University Hospitals NHS Trust, Hucknall Road, Nottingham NG5 1PB, UK; 8 Department of Pharmacology, Weill Cornell Medicine, New York, NY 10065, USA; 9 Biodiscovery Institute, School of Veterinary Medicine and Science, University of Nottingham, Nottingham NG5 1PB, UK

**Keywords:** esophageal adenocarcinoma, cancer-associated fibroblasts, phosphodiesterase type 5 inhibitors, chemotherapy, preclinical models

## Abstract

The chemotherapy resistance of esophageal adenocarcinomas (EACs) is underpinned by cancer cell extrinsic mechanisms of the tumor microenvironment (TME). We demonstrate that, by targeting the tumor-promoting functions of the predominant TME cell type, cancer-associated fibroblasts (CAFs) with phosphodiesterase type 5 inhibitors (PDE5i), we can enhance the efficacy of standard-of-care chemotherapy. In *ex vivo* conditions, PDE5i prevent the transdifferentiation of normal fibroblasts to CAF and abolish the tumor-promoting function of established EAC CAFs. Using shotgun proteomics and single-cell RNA-seq, we reveal PDE5i-specific regulation of pathways related to fibroblast activation and tumor promotion. Finally, we confirm the efficacy of PDE5i in combination with chemotherapy in close-to-patient and *in vivo* PDX-based model systems. These findings demonstrate that CAFs drive chemotherapy resistance in EACs and can be targeted by repurposing PDE5i, a safe and well-tolerated class of drug administered to millions of patients world-wide to treat erectile dysfunction.

## Highlights

• Cancer-associated fibroblasts (CAFs) drive EAC resistance
• Phosphodiesterase type 5 inhibitors (PDE5i) target the tumor-promoting function of CAFs
• PDE5i enhance the efficacy of chemotherapy in 3D-tumor models of EAC
• A randomized trial of PDE5i with chemotherapy in esophageal cancer is now required

## Graphical abstract

**Figure:**
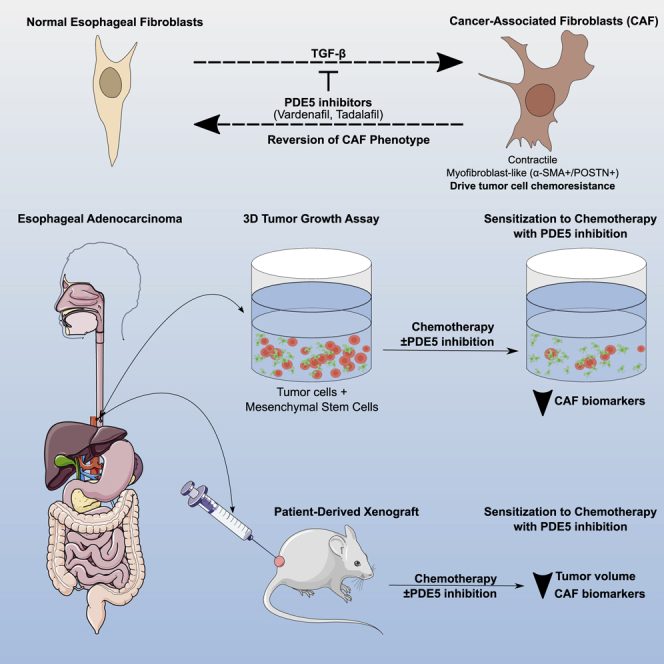

## Teaser

Resistance to standard-of-care chemotherapy in esophageal adenocarcinoma is dependent on cancer cell extrinsic mechanisms of the tumor microenvironment. Sharpe et al. show that repurposing PDE5 inhibitors to target the tumor-promoting function of cancer-associated fibroblasts enhances the efficacy of chemotherapy in 3D-tumor models

## Introduction

Esophageal adenocarcinoma (EAC) is usually lethal. Most patients present with late-stage disease, and for those amenable to potentially curative treatments, 5-year survival is 50% at best. Randomized controlled trials (RCTs) confirm a survival advantage for neoadjuvant chemotherapy with or without radiotherapy, but this benefit is restricted to a minority of patients. For the majority, neoadjuvant treatments are ineffective, are morbid, and delay definitive surgery.^1^, ^2^, ^3^

Large-scale genome-sequencing studies have detailed the genetic landscape of EAC and identified potential molecular targets. These data reveal a highly complex tumor with driver gene mutations present in non-malignant precursor lesions that never progress to cancer, suggesting that drivers of disease development and progression may lie within the tumor microenvironment.^4^, ^5^, ^6^, ^7^, ^8^

We have previously reported that activated cancer-associated fibroblasts (CAFs) influence outcome in EAC and the biological properties of esophageal CAFs that promote tumor progression.^9^^,^^10^ CAFs have also been shown to influence the immune cell infiltrate and response to chemotherapy in a range of tumors.^11^, ^12^, ^13^ In general, the tumor-promoting properties of CAFs have been associated with the alpha-smooth muscle actin (α-SMA)-positive, activated myofibroblast phenotype observed in cancer, fibrosis, and wound healing.^14^, ^15^, ^16^ CAF-targeting strategies have mostly focused on the effectors of CAF tumor promotion including cell signaling and extracellular matrix (ECM) molecules. We have been working to understand how to target the CAF phenotype itself and whether new or existing drugs can be purposed for this use.

Phosphodiesterase type 5 (PDE5) is part of a complex superfamily of hydrolases that control cAMP and cGMP levels by catalyzing their breakdown.^17^ PDE5 is widely expressed in normal tissue and many human cancers, and its inhibition results in an upregulation of cGMP, which activates several downstream pathways including protein kinase G (PKG) signaling. Downstream substrates of PKG are implicated in a variety of biological processes such as smooth muscle contraction, cell differentiation, proliferation, adhesion, and apoptosis.^18^^,^^19^ The main function of PDE5 is to control vascular tone by regulating intracellular cGMP and calcium levels.

Phosphodiesterase type 5 inhibitors (PDE5i) were first licensed to treat erectile dysfunction. More recently, high doses have been approved to treat pulmonary arterial hypertension and lower urinary tract symptoms.^20^, ^21^, ^22^, ^23^ New studies suggest repurposing PDE5i for treating conditions such as cancer or lung disease.^19^^,^^24^ PDE5i have been found to attenuate the myofibroblast phenotype of prostatic fibroblasts, suggesting that they could target the inflammatory/activated microenvironment observed in many solid tumors.^25^

We hypothesized that, in EAC, directly targeting the CAF phenotype with PDE5i would downregulate the tumor-promoting effects of CAFs and improve EAC sensitivity to conventional chemotherapy. This may improve outcomes for patients with EAC, of whom up to 80% do not respond to standard-of-care neoadjuvant treatment.^26^ Recent evidence has shown that multimodal therapies of this type have acceptable tolerability and therapeutic potential.^27^, ^28^, ^29^

In this study, we characterized PDE5 expression in the human esophagus and described the effect of PDE5i on the tumor-promoting functions of esophageal CAFs in 2D and 3D models *in vitro*. We documented changes in CAF protein expression in response to PDE5i using shotgun proteomics and applied single-cell RNA sequencing to demonstrate a phenotypic change in CAFs driven by co-culture with cancer cells and inhibited by PDE5i treatment. Finally, we moved to a validated near-patient EAC model system to assess tolerability and efficacy of PDE5i in combination with standard-of-care chemotherapy and tested the safety and efficacy of this combination in a murine model.

## Results

### Characterization of PDE5i in esophageal cancer

**Figure 1:**
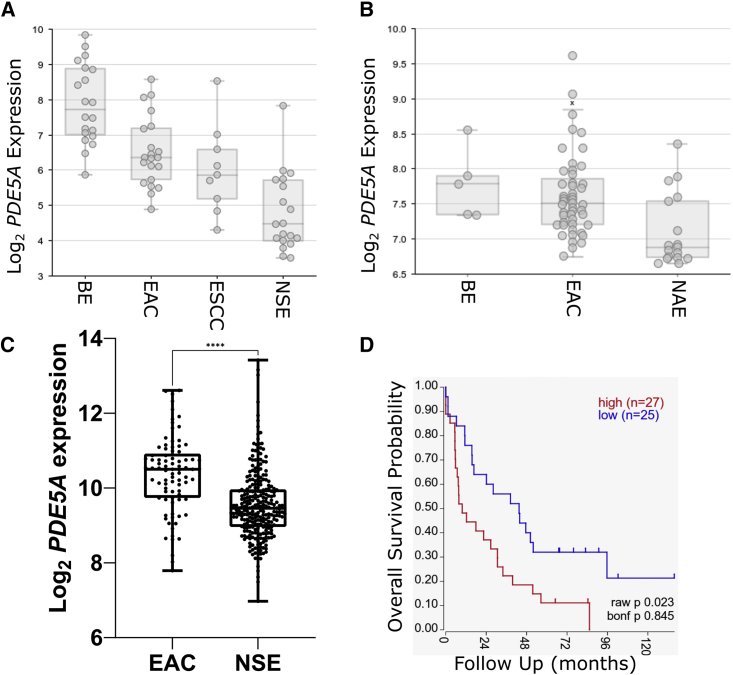
Expression of *PDE5A* in EAC patient samples compared with normal esophagus (A) Expression of *PDE5A* in the Wang et al. dataset.^30^*PDE5A* is overexpressed in Barrett’s esophagus (BE, n = 20), esophageal adenocarcinoma (EAC, n = 21), and esophageal squamous cell carcinoma (ESCC, n = 9) compared with normal squamous epithelium (NSE, n = 19). (B) Expression of *PDE5A* in the Krause et al. dataset.^31^*PDE5A* is overexpressed in BE (n = 5) and EAC (n = 48) compared with normal adjacent epithelium (NAE, n = 18). (C) Expression of *PDE5A* in RNA-sequencing data from the TCGA and GTEx projects analyzed through Xena.^35^*PDE5A* is overexpressed in EAC (n = 85) from TCGA compared with NSE (n = 269) from GTEx. ∗∗∗∗p < 0.0001. (D) *PDE5A* expression is associated with worse overall survival in EAC patients (n = 52) in Peters et al.^34^ Log2-transformed counts data were extracted and comparative analysis was performed using a Welch’s t test. Box and whisker plots represent the IQR of log2 expression of PDE5A. Central line = median, outliers are individual points outside of whiskers.

To assess the suitability of PDE5 as a target in EAC, we determined the expression of *PDE5A* in EAC relative to normal tissue, esophageal squamous cell carcinoma (ESCC), and the EAC-related pre-cursor condition Barrett’s esophagus (BE) in publicly available gene expression datasets with matching tissue samples. *PDE5A* was differentially expressed between ESCC (n = 9), EAC (n = 21), and BE (n = 20) samples compared with normal esophageal squamous epithelium (n = 19)^30^ (one-way ANOVA, p < 0.0001; Figure 1A). Specifically, *PDE5A* was significantly overexpressed in EAC, BE, and ESCC versus normal squamous epithelium (p < 0.0001). This upregulation of *PDE5A* expression in EAC and BE compared with normal adjacent esophageal epithelium was confirmed in another publicly available dataset (n = 48, 5, and 18 respectively)^31^ (Figure 1B, one-way ANOVA, p < 0.0001) and by comparing RNA-seq data from EAC samples in TCGA (n = 85)^32^ with normal esophageal squamous epithelium in the GTEx database (n = 269)^33^ (Figure 1C; Welch’s t test, p < 0.0001). Using publicly available data in R2 for EAC,^34^ we found that PDE5A expression was associated with worse overall survival ( p = 0.023; Figure 1D).

**Figure 2:**
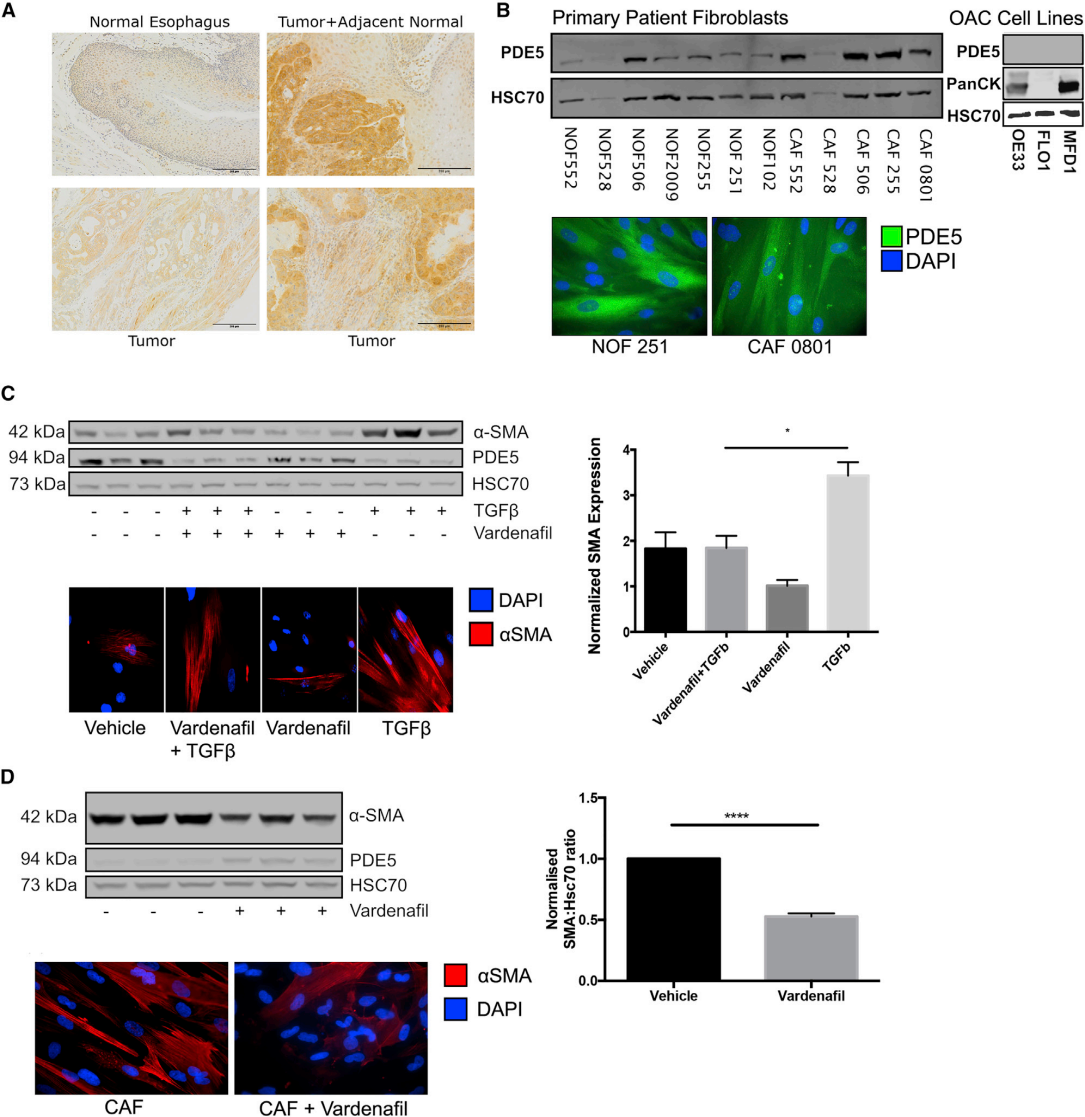
Expression of PDE5 in the esophagus and manipulation of CAF differentiation *in vitro* by PDE5i (A) PDE5 expression analysed by IHC of normal esophagus and EAC. PDE5 expression is localized to both tumor cells and the surrounding stromal tissue. Scale bars, 200 μm. (B) Western blot and ICC for PDE5 expression in three cancer cell lines (MFD-1, FLO-1, and OE33) and five matched normal (NOF)/cancer (CAF) primary esophageal fibroblasts, all normalized to Hsc70. (C) NOFs treated with TGF-β1 ± 50 μM vardenafil for 72 h. TGF-β1-treated NOFs express higher α-SMA, and vardenafil pre-treatment abrogated the TGF-β1-induced expression of α-SMA. (D) CAFs treated with 50 μM vardenafil for 72 h reduced α-SMA expression 50% by western blot and ICC. One-way ANOVA, p values: ∗p < 0.05, ∗∗∗∗p < 0.0001. Results are representative of three independent experiments. See also Figure S1.

Next, we assessed PDE5 protein expression in EAC resection specimens, normal esophageal tissue, EAC tumor cell lines, and CAFs derived from primary resected tumor tissue. In keeping with the gene expression data, PDE5 was highly and ubiquitously expressed in esophageal cancer cells and surrounding stroma compared with low expression in normal esophageal squamous epithelium (Figure 2A). In matched normal esophageal fibroblasts (NOFs) and CAFs, two commonly used EAC cell lines and a primary epithelial esophageal cancer cell line extracted in our laboratory (MFD-1),^36^ variable PDE5 expression was observed, as determined by cell sub-type. We observed the highest PDE5 expression in CAFs, with little or no PDE5 protein expression in EAC cell lines (Figure 2B; all normalized to Hsc70). Although in general a heterogeneous population, CAFs are associated with a contractile and secretory phenotype characterized by increased expression of α-SMA.^37^ This activated, myofibroblastic state in cancer is believed to be driven by cancer cell signaling and can be recapitulated by treating normal fibroblasts with TGF-β1 *in vitro*.^38^ Importantly, when one is considering any future clinical application of PDE5i in cancer treatment, it would be vital to demonstrate that not only can PDE5i revert the established CAF phenotype but also PDE5i treatment can prevent the transdifferentiation of resident NOFs to CAF. Therefore, we repeated our previous experiments to confirm that NOFs treated with TGF-β1 significantly induced α-SMA expression,^9^ but when co-treated with 50 μM vardenafil (a specific PDE5i) the increase in α-SMA expression was abolished (Figure 2C). This dose of PDE5i is high compared with its reported IC_50_ of 0.7 nM^39^ but is consistent with other studies reporting inhibition of PDE5 to target myofibroblast differentiation in fibroblast cultures with micromolar-scale concentrations of vardenafil,^25^^,^^40^^,^^41^ likely reflecting the strong myofibroblastic phenotype observed when grown in *ex vivo* culture conditions. Having established the potential of PDE5i to prevent NOF transdifferentiation *in vitro*, we explored the possibility that PDE5i could suppress α-SMA expression in CAFs. After 72 h of culture with vardenafil, CAFs reduced α-SMA expression by over 50% (p < 0.01; Figure 2D). The on-target effects of PDE5i were confirmed by observing appropriate decreases in PDE5 and α-SMA protein expression in response to PDE5 siRNA (Figures S1A–S1C). Importantly for potential *in vivo* applications, we found that daily dosing of PDE5i produced significant α-SMA downregulation compared with a single application of PDE5i-containing medium 72 h before analysis (Figure S1D). After withdrawal of PDE5i, α-SMA expression returned to pre-treatment levels within 72 h (Figure S1E).

### PDE5 inhibition reduces fibroblast contraction and esophageal cancer cell invasion *in vitro*

**Figure 3:**
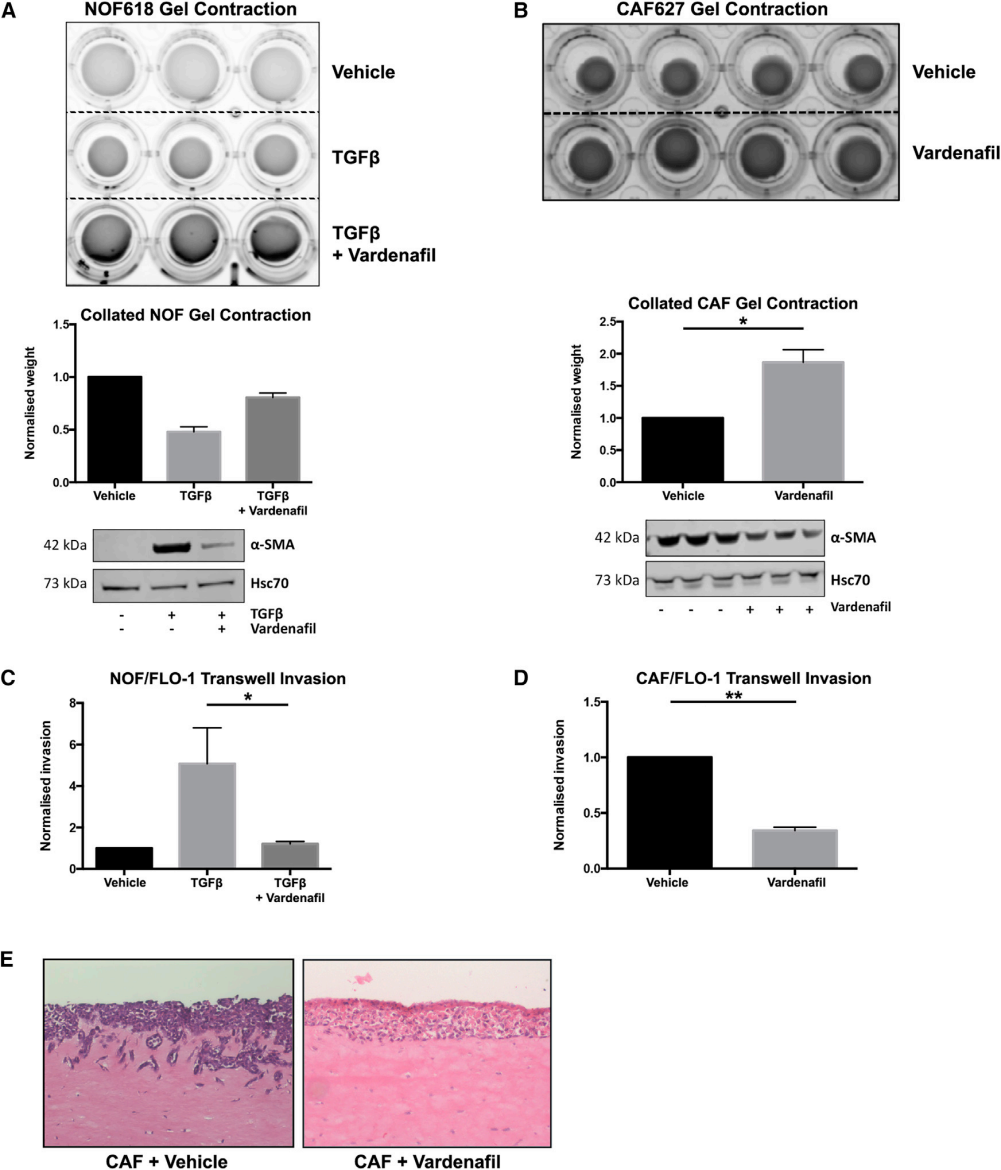
Inhibition of CAF-associated activity *in vitro* by PDE5i (A) Fibroblast contraction analyzed by NOFs treated with TGF-β1 ± 50 μM vardenafil embedded in collagen-1 gel. TGF-β1 treatment induced α-SMA expression and gel contraction in normal fibroblasts, and co-treatment with vardenafil inhibited the upregulation of α-SMA and reduced gel contraction by 50%. (B) CAF contraction was analyzed by collagen-1 gel contraction ± 50 μM vardenafil. Α-SMA expression and collagen-1 gel contraction is 2-fold greater in untreated CAFs compared with vardenafil- treated CAFs. (C and D) Cancer cell invasion was analyzed by transwell assays using conditioned medium from fibroblasts as the chemoattractant. (C) Normal fibroblasts ± TGF-β1 ± vardenafil and TGF-β1-treated NOFs promoted invasion of FLO-1 cells with a 5-fold induction; this induction was abrogated by vardenafil treatment. (D) CAFs treated with vardenafil reduced invasion of FLO-1 cells by 60%. (E) Organotypic co-culture of FLO-1 cells and CAFs also showed inhibition of invasion when treated with vardenafil. One-way ANOVA, p values: ∗p < 0.05, ∗∗p < 0.01. Results are representative of three independent experiments. See also Figure S1.

The expression of α-SMA is characteristic of the myofibroblast phenotype but does not necessarily indicate functional capacity. To test the hypothesis that PDE5i treatment of myofibroblasts could interfere with known tumor-promoting functions, we performed a series of *in vitro* experiments to assess ECM contraction and the promotion of cancer cell invasion. NOFs were embedded in collagen-1 gels after being treated with TGF-β1 alone or with TGF-β1 + vardenafil for 72 h. Fibroblasts that were treated with TGF-β1 significantly increased both α-SMA expression and collagen-1 gel contraction, but after pretreatment with vardenafil the induction of α-SMA and gel contraction was substantially and consistently reduced (Figure 3A). CAFs express high levels of α-SMA and induce collagen-1 gel contraction. Treatment with vardenafil significantly reduced α-SMA expression and collagen-1 gel contraction in CAFs (Figure 3B). Next, we assessed the ability of conditioned medium taken from fibroblast cultures to promote cancer cell invasion in transwell invasion assays under a variety of conditions, as previously described.^9^ The conditioned medium from TGF-β1-treated NOFs promoted five times more invasion of EAC cells than conditioned medium from vehicle-treated NOFs, whereas when vardenafil was added to TGF-β1 treatment of NOFs, the resulting conditioned medium did not promote invasion (Figure 3C). Similarly, vardenafil-treated CAF-conditioned medium induced significantly less cancer cell invasion than vehicle-treated CAF-conditioned medium (Figure 3D). Similar observations were made using PDE5 siRNA (Figure S1). This finding was reproduced in the more physiologically relevant organotypic co-culture model, where we observed that vardenafil-treated CAFs had lost their ability to promote cancer cell invasion compared with vehicle-treated CAF (Figure 3E). These findings suggested that, *in vitro,* PDE5i treatment was able to suppress both the transdifferentiation of NOFs and the tumor-promoting characteristics of CAF that we had previously observed.^9^

### Proteomic analysis of fibroblasts treated with vardenafil or PDE5 siRNA identifies modulation of major pathways associated with cancer promotion

In keeping with previous reports on benign disease,^25^ we established the ability of PDE5i to ameliorate some of the tumor-promoting functions of TGF-β1-driven, activated esophageal fibroblasts *in vitro*. To explore the cellular events responsible for these effects, we took a whole-proteome-based approach. We have previously demonstrated the benefits of this approach to identify pathways and participating proteins that may provide novel insight into the tumor-promoting properties of CAFs.^42^

**Figure 4:**
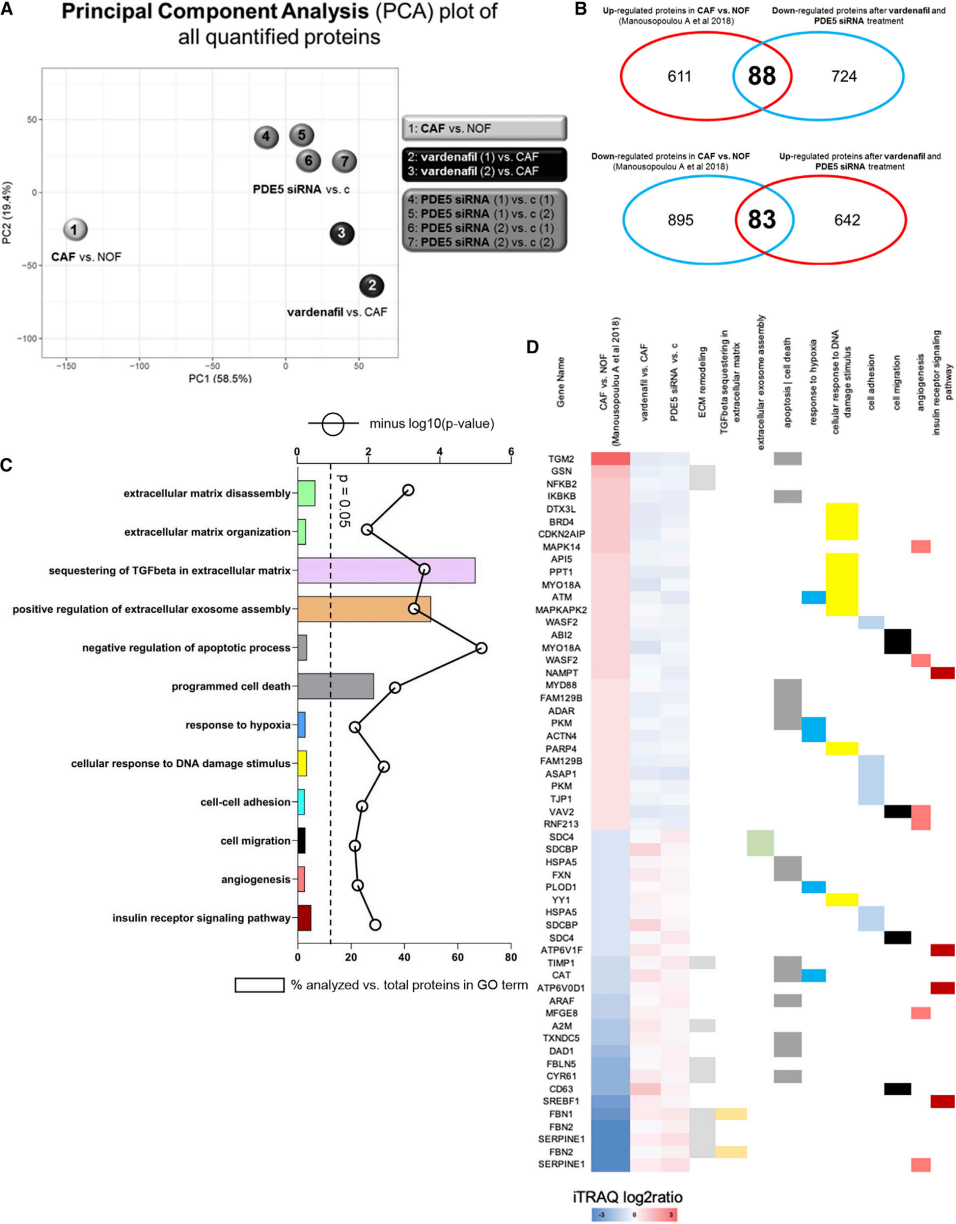
Effects of PDE5 Inhibition or siRNA on the proteomic profile of CAFs (A) Principal component analysis of all quantified proteins showed that vardenafil-treated CAFs clustered together with PDE5 siRNA-treated CAFs compared with vehicle-treated CAFs. (B) Comparison of DEPs in CAFs treated with vardenafil or PDE5 siRNA with a previously published dataset of DEPs in CAFs versus NOFs,^42^ identified 83 proteins down-regulated in CAFs versus NOFs but became up-regulated in CAFs following treatment with vardenafil or PDE5 siRNA. Conversely, 88 proteins were up-regulated in CAFs versus NOFs but became down-regulated in CAFs following treatment with vardenafil or PDE5 siRNA. (C) GO analysis using DAVID of the 171 proteins that reversed their trend of modulation following treatment with vardenafil or PDE5 siRNA compared with CAFs. (D) Proteins mapping to the respective GO terms.

Proteomic analysis was carried out on a representative NOF/CAF patient-matched pair. The CAFs were treated with vehicle (negative control), vardenafil (PDE5i), PDE5 siRNA (positive control), and negative control siRNA and total protein expression assessed by quantitative proteomic profiling. To examine the effects of vardenafil or PDE5i siRNA treatment on the global proteomic profile of CAFs, we considered the following log2 ratios: PDE5i versus CAF vehicle, PDE5 siRNA versus siRNA negative control, and CAF vehicle versus NOF. In total, 8,118 proteins were quantified across all analyzed samples (peptide level FDR < 0.05; Table S1). Principal component analysis of all quantified proteins showed that vardenafil-treated CAFs clustered together with PDE5 siRNA-treated CAFs compared with vehicle-treated CAFs (Figure 4A). Since their global proteomic profiles were similar, we considered PDE5i versus CAF vehicle and PDE5 siRNA versus siRNA negative control as one group and performed a one-sample t test to identify differentially expressed proteins (DEPs) following treatment with vardenafil or PDE5 siRNA. In total, 812 proteins were found to be up-regulated and 725 down-regulated in CAFs treated with vardenafil or PDE5 siRNA compared with their respective controls (Table S2). In order to identify which of these proteins reflected the amelioration of the CAF phenotype following vardenafil or PDE5 siRNA treatment, we compared the DEPs in CAFs treated with vardenafil or PDE5 siRNA to our previously published dataset of DEPs in CAFs versus NOFs.^42^ Using this approach, we identified 83 proteins that were down-regulated in CAFs versus NOFs but became up-regulated in CAFs following treatment with vardenafil or PDE5 siRNA (Figure 4B). Conversely, we identified 88 proteins that were up-regulated in CAFs versus NOFs but became down-regulated in CAFs following treatment with vardenafil or PDE5 siRNA (Figure 4B) (Table S3). We then performed gene ontology analysis for those 171 proteins that reversed their trend of modulation following treatment with vardenafil or PDE5 siRNA compared with CAFs. ECM organization (p = 0.01), ECM disassembly (p = 0.0008), sequestering of TGF-β in ECM (p = 0.0003), regulation of extracellular exosome assembly (p = 0.0005), cell-cell adhesion (p = 0.01), cell migration (p = 0.02), DNA damage response (p = 0.003), regulation of apoptosis (p < 0.0001), programmed cell death (p = 0.002), angiogenesis (p = 0.02), response to hypoxia (p = 0.02), and insulin receptor signaling pathway (p = 0.006) were significantly over-represented gene ontology (GO) terms (Figure 4C). These are all major pathways associated with the cancer-promoting properties of fibroblasts and identified in our previous studies of EAC fibroblasts.^42^ To provide additional granularity, proteins exhibiting the most significant changes in expression in response to PDE5i treatment have been represented on a heatmap with the corresponding GO term highlighted (Figure 4D).

In summary, these findings suggest that vardenafil is specific for PDE5 inhibition in *ex-vivo* esophageal CAFs and leads to down-regulation of established cancer-promoting CAF pathways.

### Single-cell RNA sequencing reveals suppression of activated CAF phenotypes in MFD-1/CAF co-cultures treated with a PDE5 inhibitor

To this point, experiments had focused on understanding the specificity of PDE5i in prevention of fibroblast transdifferentiation and the functional/phenotypic effects of PDE5i on CAFs. To be useful as a potential CAF-targeting treatment in cancer, these effects would need to be retained in the presence of cancer cells and be able to overcome any cancer cell-derived CAF-promoting signaling. To explore this, we took a single-cell whole-transcriptomic approach using droplet-based microfluidics and single-cell RNA sequencing (DropSeq) to analyze the gene expression of individual CAFs and esophageal cancer cells in direct co-culture.^43^ CAFs were grown in isolation or in co-culture with the esophageal cancer cell line, MFD-1.^36^ Cells were treated with vardenafil for 72 h before analysis, where indicated. This model was used to look at the transcriptional regulation of both cancer cells and CAFs in the presence of PDE5 inhibition.

**Figure 5:**
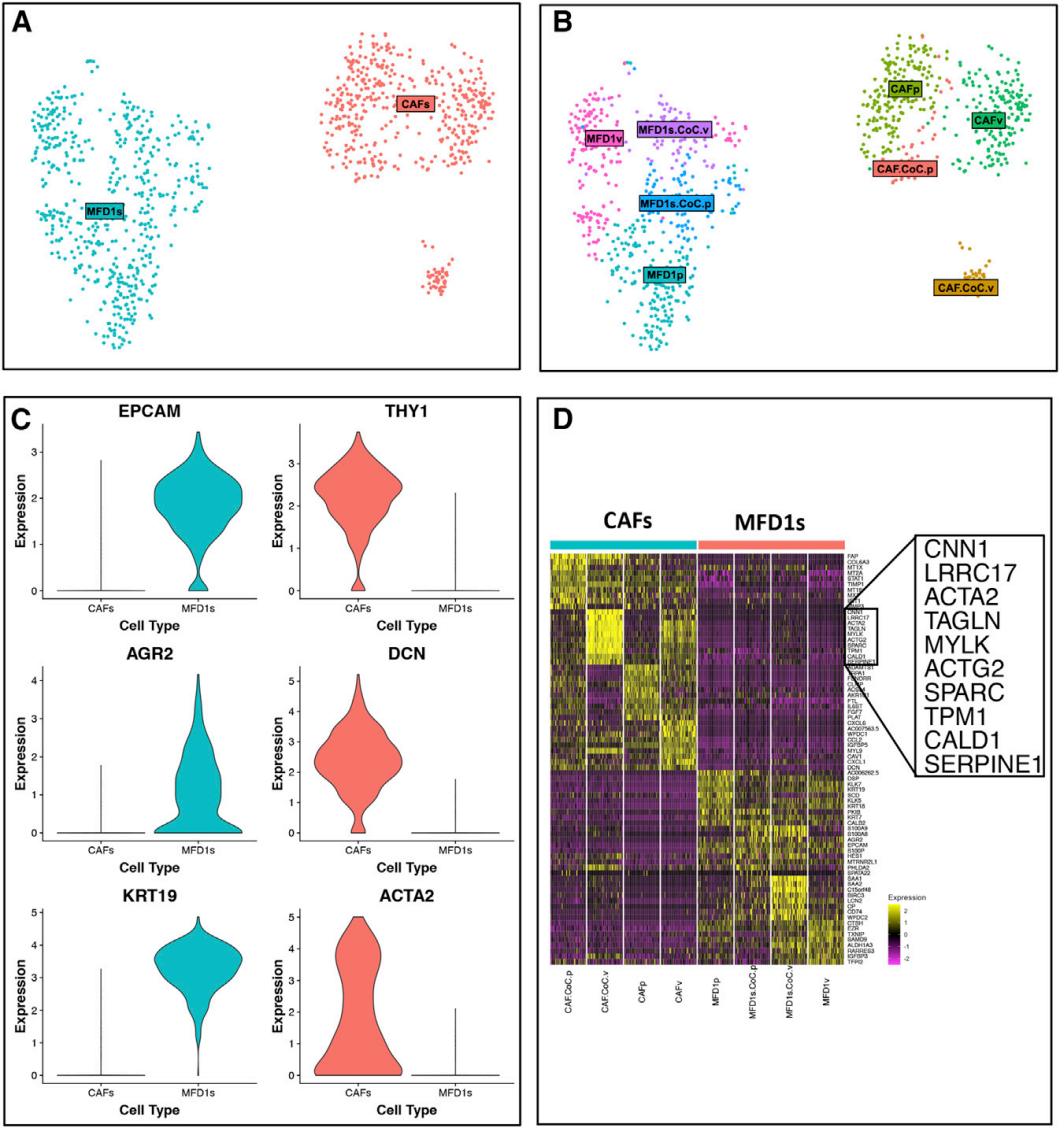
Single-cell RNA sequencing of co-cultured EAC cell lines and CAFs treated with PDE5i (A) Cluster analysis of MFD-1 and primary CAFs based on differentially expressed genes from single-cell RNA sequencing analysis (n = 1,122 cells). First, cells were clustered as either cancer cells (MFD-1, blue) or fibroblasts (red). (B) Further cluster analysis showed CAFs treated with PDE5 inhibitor vardenafil (CAFp) clustered separately from vehicle-treated CAFs (CAFv). PDE5i-treated co-cultured CAFs clustered with all other CAF cultures, whereas vehicle-treated CAFs co-cultured with MFD-1 show a phenotypic shift and clustered separately. (C) Violin plots showing typical gene expression profiles for fibroblasts (THY1, DCN, ACTA2) or epithelial cells (KRT8, KRT18, EPCAM) show good separation between the clusters in A. (D) Heatmap shows the top differentially expressed genes between treatment groups. CAFs and MFD-1 cells have the most distinct gene expression profiles, and although PDE5i treatment and co-culture both affect gene expression of both cell types, CAFs were most affected under both conditions.

Unsupervised clustering produced two broad clusters of cells, identified as either MFD-1 (cancer cells) or CAFs, as defined by their transcriptomic profiles (Figure 5A). Within these clusters a further eight sub-clusters were identified (Figure 5B). In general, the cells clustered on the basis of their cell type and within those clusters on their culture conditions.

To characterize the individual sub-clusters of cell phenotypes, differential gene expression analysis was performed using Seurat’s FindAllMarkers function with a log fold change cut-off of 1 and otherwise default settings. Canonical marker genes differentially expressed between populations of CAFs (Thy-1/CD90, decorin [DCN], smooth muscle actin [ACTA2]), and MFD-1s (epithelial cell adhesion molecule [EpCam], anterior gradient protein 2 homolog [AGR-2], and keratin-19 [KRT19]) are shown in Figure 5C.

The most striking finding from this experiment was the differential expression pattern of CAFs grown in different culture conditions. All CAFs from monoculture were similar in their transcriptomic profiles to each other (Figure 5B; CAFp and CAFv). Importantly, the PDE5i-treated CAFs from monoculture (CAFp) and co-culture (CAF.CoC.p) clustered together, whereas the CAFs co-cultured with MFD-1 in the absence of PDE5i formed a distinct cluster (Figure 5B, CAF.CoC.v). This cluster was markedly different to those from the monoculture with vehicle treatment and all other CAFs, indicating a phenotypic change in CAFs driven by co-culture with cancer cells and inhibited by PDE5i treatment.

The gene expression that defined the transcriptome of CAFs in co-culture is highlighted in Figure 5D. All are associated with the activated/myofibroblast phenotype of CAFs. These data demonstrate that CAFs adopt a myofibroblastic phenotype in co-culture with cancer cells *in vitro* and that this process can be inhibited by treatment with PDE5i, despite the presence of cancer cells, resulting in down-regulation of myofibroblast genes such as ACTA2 (α-SMA), myosin light-chain kappa (MYLK), osteonectin (SPARC), and transgelin (TAGLN) (Figure 5D).

### 3D co-culture models of close-to-patient cancer cells and human mesenchymal stem cells reveal that PDE5 inhibition increases the efficacy of chemotherapy in EAC

**Figure 6:**
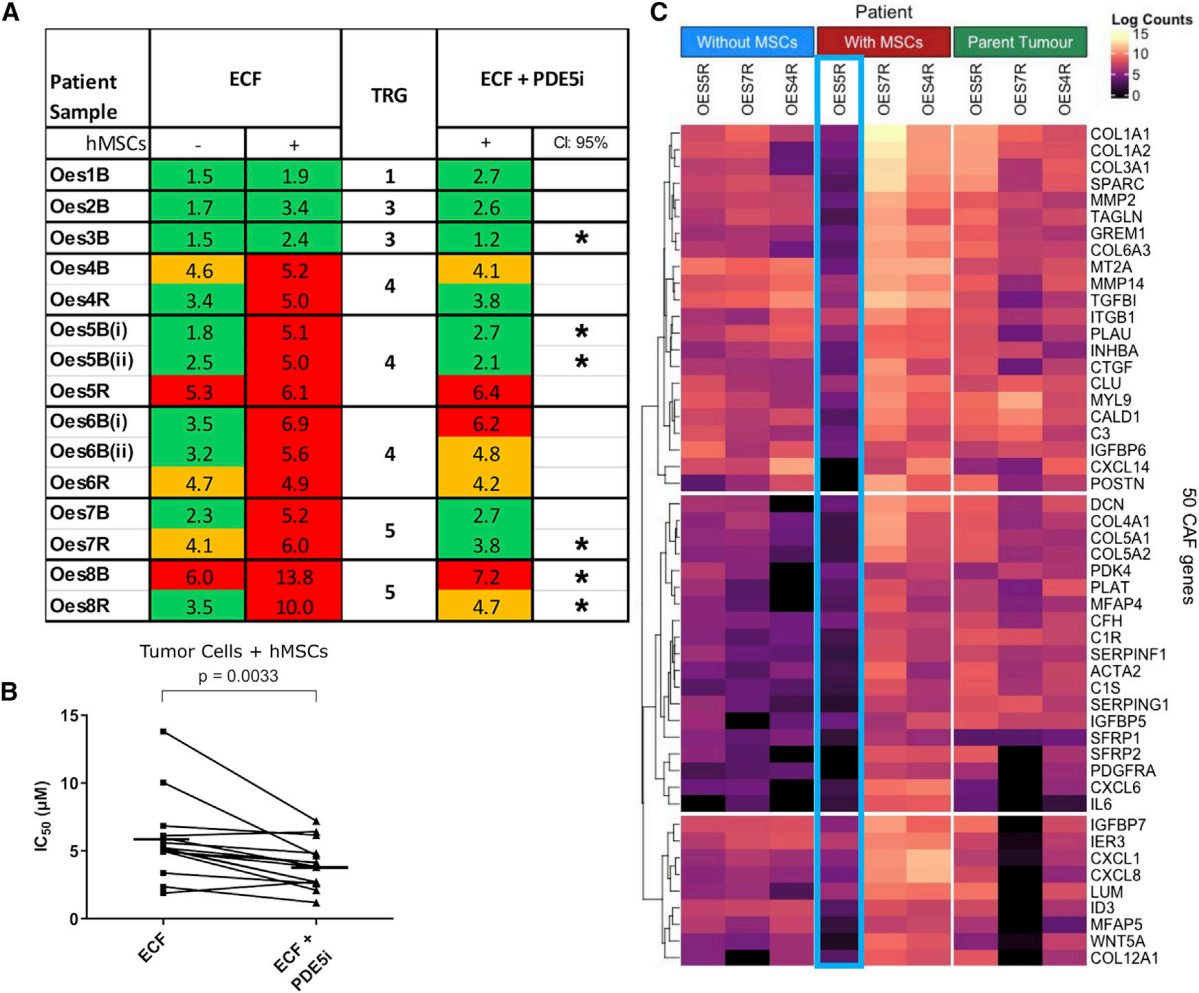
Modeling of patient response to chemotherapy with PDE5i using 3D-TGA. Sensitivity of close-to-patient cells was determined in 3D-TGA, with and without mesenchymal cell co-culture, after 4-day exposure to ECF and vardenafil (PDE5i) drug combinations (A) Viability curves were generated and IC_50_ values determined for a cohort of EAC patients’ ECF-treated 3D-TGAs, with (+) and without (−) hMSC support and the addition of PDE5i (n = 15 patient samples from 8 patients). The patient cancer cell clusters were classified as sensitive (green), borderline (orange), or resistant (red) by comparison of IC_50_ values with the mean peak serum concentrations achieved in patients at the doses used in UK clinical practice. This is marked with an asterisk, where the IC_50_ drop is significant (CI > 95%, p < 0.05). Tumor regression score (TRG) denotes the chemotherapy response of the patient’s tumor clinically (TRG1-3, sensitive; 4–5, non-responsive). (B) Overall sensitivity of all the EAC patient samples co-cultured with hMSCs was determined for assays with and without the addition of PDE5i to ECF chemotherapy. Horizontal lines represent mean IC_50_s. (C) Gene expression profiling of CAF phenotypes by bulk RNA-seq of 3D-TGA samples from three patients (Oes4R, Oes5R, and Oes7R) with and without hMSC support, and corresponding parent tumors. Heatmap showing expression of 50 well-recognized CAF marker genes. Whereas Oes4R and Oes7R upregulated CAF marker gene expression when hMSC support was added, Oes5R failed to do so (column outlined in blue). See also Figure S2.

Having established that the myofibroblast phenotype can be suppressed in 2D culture by using high concentrations of PDE5i, we sought to confirm our findings in more representative pre-clinical models. Pre-treatment biopsy tissue can be used to grow a patient’s own cancer epithelial cells *ex vivo* in the recently established 3D tumor growth assay (3D-TGA), providing a platform for near-patient drug sensitivity assessment.^44^ We used this platform to test the effect of PDE5i on EAC tumor cell sensitivity to platinum-based triplet (ECF) chemotherapy using 15 samples from eight different patients, with drug combinations at human tissue-relevant concentrations. Human mesenchymal stem cells (hMSCs) were included in the 3D-TGA, and these take up a myoCAF phenotype in culture and may be a source of CAFs in cancer.^45^, ^46^, ^47^ In keeping with our previously published data,^44^ only when cancer cells were grown with stromal support (hMSC co-cultures) did the models accurately predict the tumor regression grade (TRG)^48^ observed in the patients from whom the cancer cells had been taken (Figure 6A, lack of response to ECF represented by red). PDE5i treatment did not have toxic effects on hMSCs or EAC tumor cells, either alone or together in the 3D-TGA (Figure S2). As previously observed, chemotherapy IC_50_ was increased in co-culture compared with the no-hMSC controls (monoculture). There was no change in IC_50_ with the addition of PDE5i to ECF in monoculture, but the addition of PDE5i in co-culture resulted in a significant reduction in the IC_50_ (p = 0.0033; Figures 6A and 6B), to a level similar to or less than the mean peak serum concentration used in clinical practice. A PDE5i-mediated reduction in chemo-resistance was found to be patient specific, with the size of the PDE5i chemo-sensitizing effect varying between patients (Figure 6A); there was a trend toward a reduction in IC_50_ seen in 12 of the 15 samples and a statistically significant (CI > 95%, p < 0.05) reduction in the IC_50_ chemo-resistance in six of these samples. This suggests that ECF-resistant tumors could become sensitive to standard-of-care chemotherapy with the adjunctive administration of PDE5i. In order to understand the apparent lack of response to adjunctive PDE5i in some of the 3D-TGA models, we performed bulk RNA-seq on three patients’ tumor samples and corresponding 3D-TGAs with and without hMSCs (OES4R, 5R, and 7R). The RNA-seq data from the 3D-TGAs was analyzed for the presence of canonical myoCAF genes to enable a direct comparison with the native tumor of origin. In OES4R and 7R, we saw up-regulation of myoCAF genes in co-culture (Figure 6C). In both, ECF resistance was induced in co-culture, and resistance was reduced with PDE5i. In the non-responder (5R), we did not detect myoCAF genes up-regulated in co-culture with hMSCs (Figure 6C), and the 3D-TGA with PDE5i remained resistant to ECF (Figure 6A). This suggests either that the hMSCs did not adopt a myoCAF phenotype or that they were absent and that 5R was resistant to ECF independently of stromal support.

In 9 of 12 3D-TGA cultures from the five patients whose tumors demonstrated a poor response to chemotherapy in the clinic (TRG 4/5), the addition of PDE5i resulted in complete or partial response to ECF at doses equivalent to those observed in humans (Figure 6A), and all five patients had at least one 3D-TGA with a response. Our near-patient experiments suggest that up to 75% (9/12) of resistant tumors could be rendered sensitive to standard chemotherapy by targeting myoCAFs with PDE5i.

### PDE5i is safe and effective in combination with standard-of-care chemotherapy in esophageal patient-derived xenograft (PDX)-bearing mice

Before considering a human trial of PDE5i in EACs, we performed a dose-escalation study to assess potential serious toxicity of combining chemotherapy with PDE5i and an efficacy study in a PDX mouse model supplemented with human stromal support. The PDX was developed with esophageal cancer tissue taken from patient tumor sample Oes7R. This was chosen as it was resistant to chemotherapy clinically and in the 3D-TGA but was responsive to adjunctive PDE5i. Since human stroma is lost in PDX models over time, hMSCs were incorporated at passage to maintain a human stroma. A dose-finding study was initially performed with epirubicin, cisplatin and capecitabine (the oral equivalent of 5-FU) (ECX) in non-tumor-bearing mice (n = 3), using maximum doses equivalent to those used in humans (Table S4). This revealed peak tolerable doses of ECX that were 50% of the human equivalent doses (Figure S3). Next, dose escalation studies for PDE5i were carried out in four groups of PDX-bearing mice (no treatment [n = 2], ECX alone [n = 2], ECX + PDE5i [vardenafil, n = 3], and ECX + PDE5i [tadalafil, n = 3]). PDE5i dose increases were carried out in three phases in combination with a static dose of ECX (as previously determined). No adverse side effects (e.g., weight loss or lowered threshold of ECX tolerability) were reported for maximum doses of either vardenafil or tadalafil. Representative sections of native spleen, liver, aorta, and heart were assessed and showed no gross morphological differences between groups (Figure S4), suggesting no deleterious effects with the addition of PDE5i. However, some slight weight loss in the ECX group was attributed to the use of epirubicin. Given this concern, and in line with animal welfare best practice, we conducted an additional study with cisplatin and capecitabine only (CX) at 75% of the doses used in the previous study, which was well tolerated.

**Figure 7:**
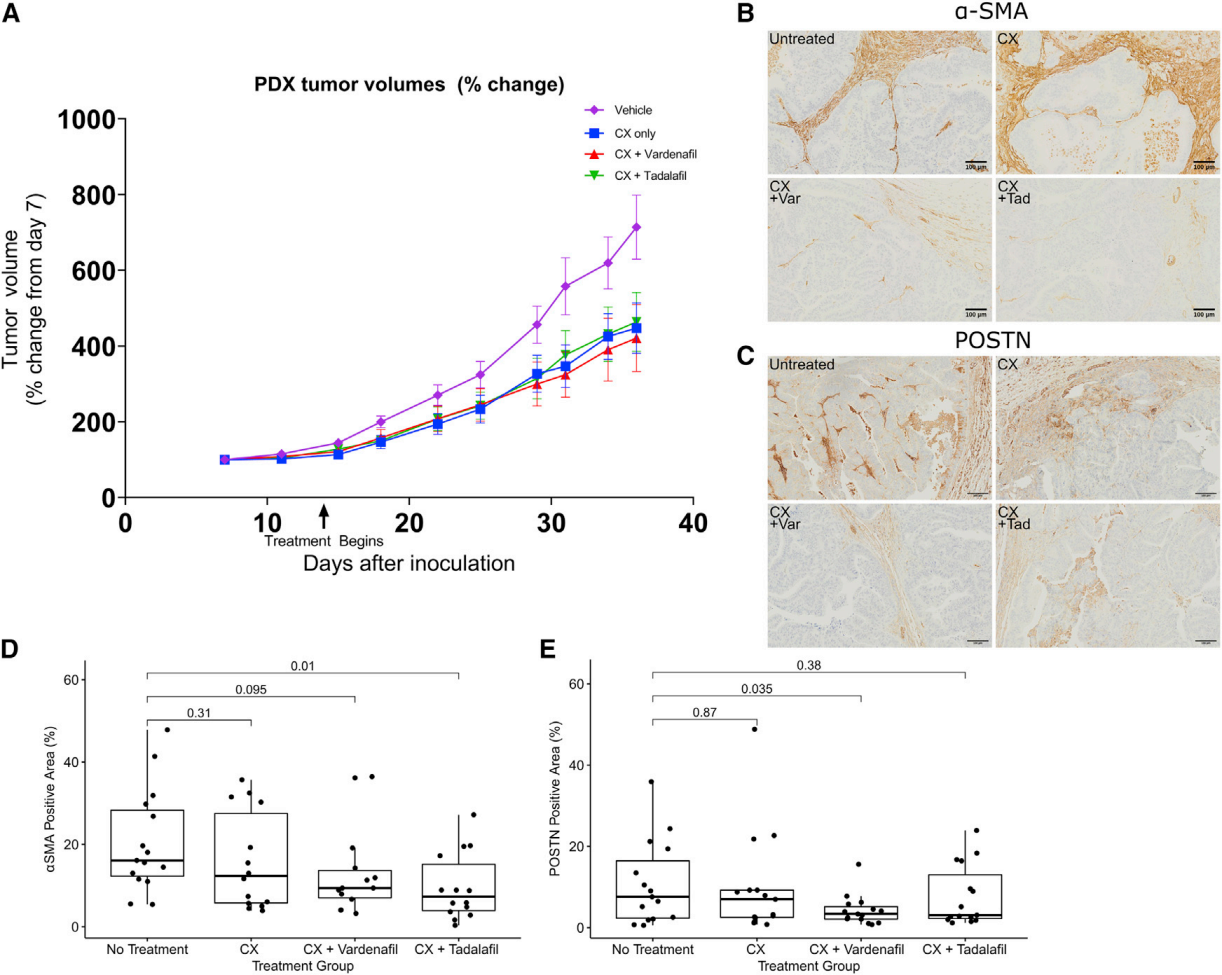
Efficacy study of PDX mouse models treated with chemotherapy alongside PDE5i (A) Tumor volumes of PDX models treated with saline for injection (vehicle), CX alone, or CX + PDE5i vardenafil (Var) or tadalafil (Tad) (n = 15 per treatment arm). (B and C) Example images of Untreated, CX, CX + Vardenafil, and CX + Tadalafil treatment arms in mouse PDX models are presented: IHC stained with (B) anti-α-SMA and (C) anti-periostin antibodies. (D and E) CAF differentiation was quantified by thresholding of digital whole-slide images from PDX models to measure total area stained for α-SMA (D) and periostin (E). See also Figures S3 and S4 and Table S4. Box and whisker plots represent the IQR of log2 expression of PDE5A. Central line = median, outliers are individual points outside of whiskers.

Having identified the most appropriate standard-of-care regimen for our PDX models, we carried out an efficacy study with a larger cohort of mice in four groups as before (n = 15/group), with one group receiving saline for injection and the others receiving a static dose of CX with or without three cycles of PDE5i vardenafil or tadalafil (see STAR Methods). A significant reduction in tumor volume was observed in the CX, CX + Vardenafil, and CX + Tadalafil groups (Figure 7A, two-way ANOVA, p values of < 0.003, < 0.0001, and < 0.007, respectively). Comparison of the tumor volume in the 4 groups on the final day of the study revealed that only the CX + Vardenafil group was significantly reduced compared with vehicle control (Kruskal-Wallis test, p = 0.04), demonstrating the stronger effect of CX + Vardenafil compared with ECX treatment alone or CX + Tadalafil. We also assessed effects of PDE5i treatment on CAF differentiation *in vivo* by conducting immunohistochemistry (IHC) for the CAF markers α-SMA and periostin in PDX tumor sections. We quantified changes in CAF markers by scanning these slides and digitally assessing the proportion of tissue stained by IHC relative to total tissue area in these treatment groups. CX treatment alone had no effect on α-SMA or periostin expression in PDX tumors compared with untreated mice (p = 0.31 and 0.87, respectively), but both PDE5i (vardenafil and tadalafil) were observed to suppress markers of CAF differentiation in PDX tumors when combined with CX (Figures 7B and 7C). CX + Vardenafil significantly reduced the proportion of periostin-positive stroma, whereas α-SMA was affected to a lesser extent (Figures 7D and 7E, p = 0.035 and 0.095, respectively). CX + Tadalafil significantly reduced the proportion of α-SMA-positive stroma (Figures 7B and 7D, p = 0.01) but not periostin-positive stroma (Figures 7C and 7E, p = 0.38).

## Discussion

Stromal remodeling can promote cancer progression. CAFs display an activated myofibroblast phenotype and expression of α-SMA in many solid tumors is a marker of reduced disease free and overall survival.^9^^,^^38^^,^^49^, ^50^, ^51^ The tumor-promoting biology of CAFs make them a target for novel cancer therapies. In this study, we have demonstrated that PDE5 is a potential target for altering the fibroblast phenotype in EACs. Using a combination of conventional *in vitro* molecular biology techniques, and state-of-the-art proteomic and single-cell sequencing technologies, we have documented the specificity of PDE5i for fibroblasts both to prevent transdifferentiation of normal fibroblasts and to reverse the activated myofibroblast (CAF) phenotype. Finally, in a step toward a clinical trial, we have confirmed the efficacy of PDE5i in combination with chemotherapy in close-to-patient *in vitro* and *in vivo* PDX-based model systems.

Our findings are in keeping with many reports documenting the role of fibroblasts in cancer. Activated myofibroblasts are contractile and pro-invasive in EAC models.^9^ There is evidence that CAFs can protect cancer cells from chemotherapy,^52^ create an immunosuppressive environment, reduce the immune infiltrate, and alter the immune composition, allowing cancer cells to escape immune surveillance.^53^, ^54^, ^55^ By reducing the transdifferentiation of fibroblasts in cancer and by modulating the phenotype of activated CAFs, we may be able to improve overall survival by several different mechanisms: improving response to chemo/immunotherapy, increasing tumor cell recognition by the immune system, and reducing cancer cell invasion.

Several strategies have been proposed to target pro-tumorigenic CAF functions, mostly through modulating the effectors of the CAF phenotype rather than the cell state itself. These include targeting the ECM-remodeling enzymes such as the lysyl oxidase family and MMPs, or targeting CAF-derived molecular signals (e.g., CXCR4, TGF-β, HGF; reviewed in Orsulic et al.^10^).

Initial attempts to specifically target CAFs have centered on the membrane bound glycoprotein fibroblast activation protein alpha (FAP). Early promise with FAP-targeting monoclonal antibodies has not translated into clinical success (reviewed in Lindner et al.^56^). Novel mechanisms to prevent myofibroblast differentiation and CAF accumulation are now required. PDE5i might offer a compelling way forward for this purpose. Importantly, PDE5i are a safe and well-tolerated class of drug administered to millions of patients world-wide to treat erectile dysfunction, benign lower urinary tract symptoms, and pulmonary arterial hypertension.^22^^,^^57^ High-dose PDE5i show safety and efficacy for treating heart failure with reduced ejection fraction.^58^ There is a significant body of evidence supporting PDE5i use in treating a range of cancers (reviewed in Pantziarka et al.^19^). In particular, animal studies suggest that PDE5i have potent immunomodulatory activity that warrants clinical study with or without immune check-point inhibition.^59^ PDE5i are currently being tested in combination with standard-of-care and other novel treatments in a range of cancer types, including gliomas, head and neck squamous cell cancer, pancreatic cancer, and malignant melanoma.^19^ With this background, the sensible next steps for testing PDE5i in EAC are in the context of a phase I/II human clinical trial.

In summary, we provide *in vitro*, near-patient, and *in vivo* evidence for the potential role of PDE5i in treating esophageal adenocarcinoma and suggest a rationale for future human trials.

### Limitations of the study

This study is not without shortcomings. Some of the *in vitro* work has been performed with cell lines that may not represent the true *in vivo* biology of these cell types. We supplemented these findings with those of more representative close-to-patient *in vitro* models that more accurately reflect the response of EAC cells to chemotherapy. Our proteomic analysis was conducted with a single representative NOF/CAF pair, and further validation of these findings might be sensible. Finally, we have not tested PDE5i efficacy in a spontaneous EAC animal model. Unfortunately, no good model of EAC exists, meaning that the best testing-ground for PDE5i in EAC will be in humans. To mitigate this, we have used a validated near-patient PDX model to demonstrate efficacy.

## STAR★Methods

### Key resources table

**Table:** 

REAGENT or RESOURCE	SOURCE	IDENTIFIER
**Antibodies**		

Rabbit polyclonal anti-PDE5A	Abcam	Cat#ab224232
Mouse monoclonal anti-alpha-SMA	Dako	Cat#M085129-2
Mouse monoclonal anti-HSC-70	Santa Cruz Biotechnology	Cat#sc-7298
Rabbit polyclonal anti-PDE5A	Santa Cruz Biotechnology	Cat#sc-32884
Rabbit polyclonal anti-Periostin	Abcam	Cat#ab14041
AlexaFluor568 donkey anti-mouse IgG	Invitrogen	Cat#A10037
AlexaFluor488 goat anti-rabbit IgG	Invitrogen	Cat#A11034
EnVision + HRP reagents, anti-mouse	Dako	Cat#K4001
EnVision + HRP reagents, anti-rabbit	Dako	Cat#K4003

**Chemicals, peptides, and recombinant proteins**		

Vardenafil	Sigma-Aldrich	Cat#V-902
Recombinant TGF-β1	R&D Systems	Cat#240-B
DAPI	Sigma-Aldrich	Cat#D9542
Epirubicin	Sigma-Aldrich	Cat#E9406
Cisplatin	Sigma-Aldrich	Cat#232120
5-Fluorouracil	Sigma-Aldrich	Cat#F6627
Capecitabine	Sigma-Aldrich	Cat#SML0653
Tadalafil	Sigma-Aldrich	Cat#SML1877
INTERFERin	Polyplus	Cat#409-10
Matrigel	Corning	Cat#356234
pLVX-fLuc	In-House	N/A

**Critical commercial assays**		

Nextera XT DNA Library Prep Kit	Illumina	Cat#FC-131-1024
NextSeq500/550 High Output Kit (75 Cycles)	Illumina	Cat#20024906
Venor GeM Classic Kit	Minerva Biolabs	Cat#11-1250
alamarBlue® cell fluorescence assay kit	ThermoFisher Scientific	Cat#DAL1025

**Deposited data**		

Gene expression microarray	Krause et al.^31^	GEO: GSE72874
Gene expression microarray	Wang et al.^30^	GEO: GSE26886
Gene expression microarray and clinical data	Peters et al.^34^	GEO: GSE19417
TCGA RNA-Seq	Kim et al.^32^	https://www.cancer.gov/tcga
GTEx	Ardlie et al.^33^	https://www.gtexportal.org/
Mass Spectrometry Proteomics Data	This Paper	ProteomeXchange: PXD031148
3D-TGA Bulk RNA-Seq	This Paper	GEO: GSE194277

**Experimental models: Cell lines**		

FLO-1	ECACC	Cat#11012001
OE33	ECACC	Cat#96070808
MFD-1	Previously generated in our lab: Garcia et al.^36^	N/A
human bone marrow-derived mesenchymal stem cells (hMSC)	ScienCell Research Laboratories	Cat#7500

**Experimental models: Organisms/strains**		

Mouse: CD-1 nude: Crl:CD1-*Foxn1*^*nu*^	Charles River UK	N/A

**Oligonucleotides**		

PDE5 siRNA 1: 5′-CCAGUGCUCAAGACUCUUGtt-3′, 3′-ctGGUCACGAGUUCUGAGAAC -5′	Ambion	Cat#137131
PDE5 siRNA 2: 5′-GCAAGCUAUUUUAGCUACAtt-3′, 3′-ttCGUUCGAUAAAAUCGAUGU-5′	Ambion	Cat#137133
Negative control siRNA #1	Ambion	Cat#4457287

**Software and algorithms**		

R2: Genomics Analysis and Visualization Platform	Academic Medical Center, University of Amsterdam	http://r2.amc.nl/
UCSC Xena	Goldman et al.^35^	https://xenabrowser.net/
GeneAnnot	Weizmann Institute of Science	https://genecards.weizmann.ac.il/geneannot/index.shtml
ClustVis	Metsalu and Vilo^60^	https://biit.cs.ut.ee/clustvis/
DAVID	Huang et al.^61^	https://david.ncifcrf.gov/summary.jsp
drop-seq-tools v1.1.2	Macosko et al.^43^	https://github.com/broadinstitute/Drop-seq/releases
R v3.5.1 “Feather Spray”	Comprehensive R Project Network	https://cran.r-project.org/
R v4.1.0 “Camp Pontanezen”	Comprehensive R Project Network	https://cran.r-project.org/
Seurat v2.3.4	Satija et al.^62^	http://satijalab.org/seurat/
GraphPad Prism 5	GraphPad Software	N/A
GraphPad Prism 9	GraphPad Software	N/A
QuPath v0.2.0	Bankhead et al.^63^	https://qupath.github.io/
SPSS v19	SPSS	N/A

### Resource availability

#### Lead contact

Further information and requests for resources and reagents should be directed to and will be fulfilled by the Lead Contact, Prof. Tim Underwood (tju@soton.ac.uk), Professor of Gastrointestinal Surgery, School of Cancer Sciences, University of Southampton, Hampshire, UK.

#### Materials availability

This study did not generate new unique reagents.

### Experimental model and subject details

#### Cell lines

FLO-1 (ECACC), OE33 (ECACC), MFD-1 and primary cells were maintained in Dulbecco’s modified Eagles medium (DMEM, Invitrogen) or Roswell Park Memorial Institute medium (RPMI, Invitrogen) supplemented with 10% (v/v) fetal calf serum (FCS, Autogen Bioclear), 2 mM L-glutamine and 100 μg/mL penicillin/streptomycin (Invitrogen). The MFD-1 cell line is a well characterized primary esophageal cancer cell line and has been described previously.^36^

#### Primary fibroblast cultures

Tissue was collected and stored with ethical agreement and informed consent (REC: 09/H0504/66 and 18/NE/0234) at University Hospital Southampton, and fibroblasts were extracted from normal esophagus and esophageal adenocarcinoma and sub-cultured as previously described.^64^

#### Mouse models

The *in vivo* experiments were conducted under the UK Home Office Licence number PPL P435A9CF8. LASA good practice guidelines, FELASA working group on pain and distress guidelines and ARRIVE reporting guidelines were also followed.

All mice were purchased from Charles River UK. Mice were maintained in individually Ventilated Cages (Tecniplast UK) within a barriered unit, illuminated by fluorescent lights set to give a 12 hour light-dark cycle (on 07.00, off 19.00), as recommended in the guidelines to the Home Office Animals (Scientific Procedures) Act 1986 (UK). The room was air-conditioned by a system designed to maintain an air temperature range of 21 ± 2°C and a humidity of 55% ± 10%. Mice were housed in social groups, 3 per cage, during the study, with irradiated bedding and autoclaved nesting materials and environmental enrichment (Datesand UK). Sterile irradiated 5V5R rodent diet (IPS Ltd, UK) and irradiated water (Baxter, UK) was offered *ad libitum.* The condition of the animals was monitored throughout the study by an experienced animal technician. After a week’s acclimatisation, the mice were initiated with tumors as described in the dose-finding study, dose escalation study and efficacy study as described in method details.

##### Dose-finding study for ECX administration in non-tumor bearing mice

3 female CD-1 mice received escalating doses of ECX cycle in 3 phases separated by a drug holiday of 14 days to allow recovery between cycles. Doses were calculated by converting from human standard of care regimens as described in Table S4. Dosing was as follows, with the exception of one mouse that underwent a repeat of cycle 2 (50% clinical equivalent dose) due to slower recovery. Dose cycle 3 is equivalent to 75% of the clinical equivalent ECX dose.

**Table:** 

Compound	Days	Dose cycle 1 (mg/kg)	Dose cycle 2 (mg/kg)	Dose cycle 3 (mg/kg)
Epirubicin (IV)	1	3.75	7.5	11.25
Cisplatin (IP)	1, 3, 5	1.5	3.0	4.5
Capecitabine (PO)	1, 2, 3, 4, 5	50	100	150

##### Dose escalation study of ECX in patient-derived xenograft-bearing mice

10 male 8–9 week old CD-1 NuNu mice were implanted with 1x10^6^ OES127 cells re-suspended in 100 μl of Matrigel (Corning), which were developed from an esophageal adenocarcinoma resection specimen + eGFP labelled mesenchymal stem cells (MSCs) in a ratio of 2:1. The cells were generated by disaggregating from donor OES127 PDXs by a collagenase/dispase disaggregation fluid, and rotating at 37°C for 1 hour, counted and viability measured by trypan blue, before resuspending both the MSCs and PDX simultaneously in matrigel. These were injected subcutaneously into the left flank of the mice and the resulting tumours were measured twice weekly using Vernier calipers and the volumes calculated using the formula V=ab2/6, where a is the length and b is the width. A secondary dose of MSCs, this time lentivirally transduced with pLVX-fLuc, were added as a ‘boost’, 14 days after initiation, directly injected into the tumor in Phosphate Buffered Saline (Sigma, UK). Dosing commenced on day 18 post-initiation and followed the regime below, with a 14 day rest period between each cycle for observation of side effects, during which time animals were weighed daily.

Dosing regime

• Group 1 Control-no treatment
• Group 2 Epirubicin 15 mg/kg, IV, day 1 + Cisplatin 3 mg/kg, IP, days 1, 3, 5 + Capecitabine 100 mg/kg, PO, days 1, 2, 3, 4, 5 (twice daily) [ECX]
• Group 3 ECX (as above) plus Vardenafil, twice daily at:
  - 2 mg/kg (i.e. 4 mg/kg/day), PO, days 1, 2, 3, 4, 5
  - 6 mg/kg (i.e. 12 mg/kg/day), PO, days 15, 16, 17, 18, 19, 20
  - 8 mg/kg (i.e. 16 mg/kg/day), PO; days 29, 30, 31, 32, 33
• Group 4 ECX (as above) plus Tadalafil, twice daily at:
  - 2 mg/kg, (i.e. 4 mg/kg/day), PO, days 1, 2, 3, 4, 5
  - 4 mg/kg, (i.e. 8 mg/kg/day), PO, days 15, 16, 17, 18, 19, 20
  - 10 mg/kg, (i.e. 20 mg/kg/day), PO, days 29, 30, 31, 32, 33

∗IV=intravenous, IP=intraperitoneal, PO=per os, SFI=saline for injection, WFI= water for injection

As this was a dose escalation tolerability study, no power calculation was required, groups 1 and 2 having 2 mice each and groups 3 and 4 having 3 mice each. The mice were terminated between days 42-56 due to tumors approaching maximum allowable size. They were culled by cervical dislocation, tumors were dissected out and weighed, aortas, hearts, livers and spleens were also dissected out, and all were fixed in Neutral Buffered Formalin.

Due to some slight weight loss in one group (ECX) during this dose escalation study which was thought to be associated with the use of epirubicin, an additional tolerability study was carried out using cisplatin and capecitabine only, at 75% dose of those used in the Dose Escalation (Cisplatin 2.25 mg/kg, IP, days 1, 3, 5 + Capecitabine 75 mg/kg, PO, days 1, 2, 3, 4, 5) and using the PDE5is daily (instead of twice daily at lower doses). This dosing regime, which was well-tolerated was adopted for the Efficacy study (see details below).

**Table:** 

Vardenafil	Required dose	Given to mice
Low dose (cycle 1)	4 mg/kg	4 mg/kg once daily
High dose (cycle 2)	12 mg/kg	6 mg/kg twice daily (am/pm)
Maximum dose (cycle 3)	16 mg/kg	8 mg/kg twice daily (am/pm)
Tadalafil	Required dose	Given to mice
Low dose (cycle 1)	4 mg/kg	4 mg/kg once daily
High dose (cycle 2)	8 mg/kg	8 mg/kg once daily
Maximum dose (cycle 3)	20 mg/kg	10 mg/kg twice daily (am/pm)

##### Efficacy study of PDE5i and CX in patient-derived xenograft-bearing mice

60 female 7-8 week old CD-1 NuNu mice were used for PDX experiments, testing CX treatment in combination with PDE5i, vardenafil or tadalafil. Patient-derived xenografts were developed using OES127 cells and eGFP-labelled hMSCs as above. Due to loss of the human stromal compartment in such models, human mesenchymal stem cells (hMSCs) were co-implanted with the xenograft and supplemented before treatment began as before. Tumors were measured as previously detailed, and mice were weighed weekly. Tumors were also imaged weekly in the IVIS® Spectrum imaging system (PerkinElmer, MA, USA) by 2D optical imaging, with tumor measurements made using Living Image (4.3.1) software and standard open filters to assess the retention of MSCs. Prior to imaging, the mice were anaesthetised with an injectable anaesthetic combination (Anaestemine [ketamine]/Sedastart [medetomadine], Animalcare Ltd. UK) before being placed in the IVIS system and imaged on days 0, 1, 8, 15, 16, 21, 23 and 28, mice being allowed to recover from the anaesthetic with appropriate post procedural monitoring and therapy, including placing mice on a heat pad and providing fluid replacement via wet mash once awake.

On day 14 after tumor initiation, the mice were randomised into one of four groups (n=15/group) by tumor size and fluorescence. Power calculations to determine group sizes were based on One way ANOVA with 4 groups, to allow detection of a 40% effect of treatment at a power of 80%. This gave a minimum required sample size of n=10 per group but based on a potential 70% take rate (due to the tissue being PDX in origin and the implant location) sample size was increased to n=15 per group. Dosing followed the weekly cycle below for 3 weeks, with 2 days dosing in week 4 prior to termination one week after final dosing.

Dosing regime:

• Grp 1 Cisplatin 2.25 mg/kg, IP, days 1, 3, 5 + Capecitabine 75 mg/kg, PO, days 1, 2, 3, 4, 5 [CX]
• Grp 2 CX (as above) plus Vardenafil (16 mg/kg, PO) days 1, 2, 3, 4, 5
• Grp 3 CX (as above) plus Tadalafil (20 mg/kg, PO) days 1, 2, 3, 4, 5
• Grp 4 Placebo dosed control, SFI vehicle IP days 1, 3, 5 and WFI vehicle PO days 1, 2, 3, 4, 5

The mice were culled by cervical dislocation, tumors were dissected out and weighed, before half was snap frozen in liquid nitrogen, half was fixed in Neutral Buffered Formalin and processed for paraffin embedding. Data from one of the mice in Group 2 was excluded from the final analysis because it reached the maximum allowed size a week before any of the other mice in the study needed to be terminated, and thus was considered an outlier. Growth of tumors was assessed based on caliper measurements and expressed as a percentage of the pre-treatment volume for individual mice. Mean and standard error was calculated for each group and analysed by 2-way ANOVA to compare each group to the untreated group and by Kruskal-Wallis test to compare relative tumor volume between the groups at the final timepoint.

### Method details

#### Analysis of PDE5A mRNA expression levels in public datasets

*PDE5A* gene expression data from three gene expression microarray datasets^30^^,^^31^^,^^34^ were analysed in R2: Genomics Analysis and Visualization Platform (http://r2.amc.nl, date accessed: 09/07/2021) and used to generate a Kaplan-Meier survival curve within the platform. A representative probe set was chosen for PDE5A for each dataset using GeneAnnot (https://genecards.weizmann.ac.il/geneannot/index.shtml, date accessed: 09/07/2021), taking into account sensitivity, specificity, annotation quality, gene number and highest Average Present Signal (APS) of all samples that express the gene. Comparative analysis was conducted using a one-way ANOVA in R2. Optimal cutoff level of *PDE5A* expression was determined by KaplanScan (optimum survival cutoff based on gene expression in whole cohort by logrank test) and statistically significant differences in survival ascertained using R2. *P* values were corrected by Bonferroni correction.

The Xena platform was used for gene expression analysis of RNA-seq data from the TCGA and GTEx databases (https://xenabrowser.net/).^35^ For TCGA, the ESCA dataset was used and only samples identified as esophageal adenocarcinoma were included.^32^ For GTEx, samples with the identifier “esophagus – mucosa” were used, while samples labelled as “esophagus – muscularis” and “esophagus – gastroesophageal junction” were excluded.^33^ Log2-transformed counts data were extracted and comparative analysis was performed using a Welch’s T-test in GraphPad Prism 9.3.

#### SiRNA transfections

Commercially available PDE5 sequences 1, 5’-CCAGUGCUCAAGACUCUUGtt-3’, 3’-ctGGUCACGAGUUCUGAGAAC -5’ (137131, Ambion) and Sequence 2, 5’-GCAAGCUAUUUUAGCUACAtt-3’, 3’-ttCGUUCGAUAAAAUCGAUGU-5’ (137133, Ambion) were transfected into primary fibroblasts at 50% confluence (cells were seeded 24 hours before transfection, 5x10^5^/well of a 6 well plate) using INTERFERin (Polyplus), according to the manufacturer’s instructions. Cells were cultured for 72 hours to achieve optimal knockdown, and negative control 1 siRNA (Ambion) was used for experimental controls.

#### Immunohistochemistry

Optimization and staining of cohort on full face sections using rabbit polyclonal anti-PDE5 (1:250 dilution with Dako FLEX TRS High pH retrieval; ab224232, abcam), mouse monoclonal anti-α-SMA (1:200 dilution with Dako FLEX TRS High pH retrieval; M085129-2, Dako) and rabbit polyclonal anti-Periostin (1:1000 dilution with Dako FLEX TRS Low pH retrieval; ab14041, abcam) was performed on a Dako link automated staining machine according to the manufacturer’s instructions.

#### Western blotting

Antibodies used: mouse monoclonal anti-α-SMA (M085129-2, Dako), mouse monoclonal anti-HSC-70 (sc-7298, Santa Cruz) and rabbit polyclonal anti-PDE5 (SC-32884, Santa Cruz). Normal esophageal fibroblasts were pre-treated with 50 μM vardenafil (Sigma) for 1 hour before treatment with recombinant TGF-β1 (R&D Systems) for 72 hours. Cancer associated fibroblasts were treated with 50 μM vardenafil for 72 hours or 3x 50 μM vardenafil over 72 hours as indicated. Cells were harvested by trypsin digestion after an initial PBS wash before pelleting by centrifugation. Cell lysis was carried out for 15 minutes at 4°C in 50 μl RIPA buffer (0.75M NaCl, 5% NP40, 2.5% deoxycholic acid, 0.5% SDS, 0.25M Tris pH8.0). Lysates were clarified by centrifugation at 8000xg for 5 minutes. Fibroblasts or FLO-1 cells were cultured in serum free DMEM for 24 hours before the conditioned medium was harvested at 4°C, clarified by centrifugation and the supernatant concentrated using Amicon ultra-4-centrifugal 10 kDa filters (Millipore). Protein samples were quantified using the Bradford protein assay reagent. 20 μg of protein or 20 μl of concentrated cell culture medium (adjusted in SDS loading dye for cell number when conditioned medium was removed) were resolved using SDS-polyacrylamide electrophoresis and transferred to Hybond-ECL membranes (GE healthcare). Blocking and antibody incubations were done in 3% low-fat milk in PBS–0.025% Tween 20, and washes were in PBS–0.1% Tween 20. Detection of horseradish peroxidase-labelled secondary antibody was done with Supersignal (Pierce), and images were collected using a CCD camera (ChemiDoc-it® imaging system, UVP).

#### Immunofluorescence

Immunofluorescence analyses were carried out as previously described.^65^ Primary antibodies: rabbit polyclonal anti-PDE5 (ab224232, abcam) and mouse monoclonal anti-α-SMA (M085129-2, Dako), Secondary antibodies: Alexa Fluor 568 donkey anti mouse IgG and Alexa Fluor 488 goat anti rabbit IgG (A10037 and A11034 respectively, Invitrogen). Cell nuclei were counterstained with 1 μg/ml DAPI (4’,6’-diamidino-2-phenylindole, D9542, Sigma-Aldrich).

#### Gel contraction

Gel contraction assays were conducted as previously described.^16^ Briefly, 0.5x10^6^ NOFs or CAFs (± TGF-β1, +/- 72 hours vardenafil treatment) were seeded in collagen-1 gels in 24-well plates. Gels were photographed and weighed after 24 hours.

#### Transwell invasion

Transwell invasion assays were conducted as previously described,^66^ using conditioned medium from NOFs or CAFs as the chemoattractant. NOFs and CAFs were cultured in the presence of TGF-β1 or TGF-β1 + vardenafil for 72 hours before washing out and collecting conditioned medium after 24 hours. FLO-1 cells were plated in the top chamber in identical numbers. All experiments were repeated 3 times with 4 replicates per experiment. Normalization was performed to the mean value of replicate 1 for all experimental conditions and data expressed as % invasion compared to the vehicle control.

#### Organotypic cultures

Organotypic cultures were carried out as previously described.^64^^,^^66^ Briefly, CAFs were pre-treated with 50 μM vardenafil or vehicle control for 72 hours before plating in 1:1 collagen:matrigel gels at a density of 5x10^5^ cells per gel, and the next day 5x10^5^ FLO-1 cells were cultured on top of the gels. Organotypic cultures were raised onto steel grids with overlaid nylon membrane supports and cultured at the air-liquid interface overnight before treatment. Organotypic cultures were incubated for a further ten days with added Vardenafil or vehicle control before processing. Invasion was quantified and compared as previously described.^67^

#### Proteomics

##### Quantitative proteomics analysis

Primary CAFs and NOFs were treated with 50 μM vardenafil, vehicle or PDE5 siRNA for 72 hours before trypsinisation and clarification. Cells were lysed and protein extracts were quantified. One hundred ug of protein per sample was reduced, alkylated and enzymatically digested using trypsin. Peptides were labelled using the isobaric tag for relative and absolute quantitation (iTRAQ) 8-plex reagents and analysed using two-dimensional liquid chromatography and tandem mass spectrometry as reported previously.^42^^,^^49^

##### Database searching

Unprocessed raw files were submitted to Proteome Discoverer 1.4 for target decoy search using the Sequest algorithm as reported previously.^42^^,^^49^ The UniProtKB homo sapiens database which comprised 20,159 entries (release date January 2015) was utilized. FDR corrected *p-value* at the peptide level was set at <0.05. Percent co-isolation excluding peptides from quantitation was set at 50. Reporter ion ratios from unique peptides only were taken into consideration for the quantitation of the respective protein.

The iTRAQ ratios of proteins were median-normalized and log_2_ transformed. Principal component analysis of all quantified proteins was performed using ClustVis (https://biit.cs.ut.ee/clustvis/).^60^ A one-sample Student’s T-Test was performed to identify differentially expressed proteins (DEPs) in treated cells vs. their respective controls as one group. Proteins identified with at least two unique peptides and a one-sample Student’s T-Test (*p-value*<0.05) were considered differentially expressed. Gene ontology analysis was performed using DAVID (https://david.ncifcrf.gov/summary.jsp).^61^ A Fisher-exact p-value < 0.05 was considered significant.

#### Droplet barcoded single cell RNA sequencing

CAFs were grown in isolation or in co-culture with the MFD-1 cell line.^36^ Cells were treated with vardenafil for 72 hours before analysis. This model was used to look at the transcriptional regulation of both cancer cells and CAFs in the presence of PDE5 inhibition.

Cultured cells were trypsinized and resuspended in a cell suspension buffer and single cell RNA seq libraries created using DropSeq.^43^ Captured mRNA was reverse transcribed and the resulting cDNA libraries were amplified, purified and prepared for sequencing using a modified Nextera XT protocol and sequenced using Illumina NextSeq500. Sequenced reads were aligned to Genome Reference Consortium Human Build 37 (HG19) and processed using DropSeq tools 1.12 to produce a digital expression matrix where columns are cells and rows are genes, gene counts were created by counting UMIs. Clustering and differential expression analysis was performed using R version 3.5.1 (Feather Spray) and the package Seurat version 2.3.4.^62^

Raw data consisted of 1800 cells expressing 24,434 genes. After removing genes expressed in less than 5 cells and cells with less than 1500 genes we created a Seurat object with 1122 cells expressing 18,436 genes. Gene counts were log normalised and scaled to read depth and mitotic phase to remove unwanted sources of variation using Seurat’s CellCycleScoring and ScaleData functions.

The 3,554 most highly variable genes (highest log variance to mean ratio and highest mean expression) were used to perform principal component (PC) analysis. The first principal component consisted of genes known to be markers of fibroblasts and fibroblast activity, such as Decorin, vimentin and CD90 (THY1) and genes known to be markers of adenocarcinoma such as EPCAM and the cytokeratins.

The first 10 principal components were used as input to Seurat’s FindClusters function and dimensionality reduction was performed using RunTSNE. This first pass clustering produced some populations from the co-cultured cells that were expressing known markers of fibroblasts and cancer cells suggesting that they were doublets (two cells exposed to one nanobead). We identified these cells by highlighting all cells that had a scaled expression of greater than one for any of the top 30 fibroblast markers from PC 1 and any of the top 30 markers for cancer cells from the same PC. We removed 109 cells from our dataset 104 from the co-culture experiments. We then repeated the clustering steps above without the “doublet” population.

#### 3D-tumor growth assay

The full method for the establishment of close-to-patient OAC cells using a feeder layer method and subsequent growth with a stromal component in the 3D-tumor growth assay to form cancer cell clusters, which can then undergo clinically relevant *ex vivo* pharmacological assessment, has been published by our group.^44^ Endoscopic tumor biopsies (REC: 10/H0401/80) and fresh surgical specimens (REC: 08/H0403/37) were collected with informed consent from patients at Nottingham University Hospitals NHS Trust in 2014-15, and used in accordance with National Research Ethics Service approval to generate esophageal cancer cell lines as previously described.^44^ Cell lines were routinely tested for Mycoplasma with the Venor®GeM Classic kit (Minerva Biolabs, Berlin, Germany).

In this *ex vivo* study we undertook evaluation of adjunct PDE5i administration in additional to the regimen of Epirubicin, Cisplatin and 5-Fluorouracil. This is the standard of care pre- and post-operative chemotherapy regimen used for the treatment of OAC in the UK, and was administered to patients clinically in this study. No formal sample-size estimation was made because we used consecutive patient samples until a representative number of responders and non-responders to chemotherapy had been tested. Samples were allocated to experimental groups as follows, meaning each sample had its own internal control. This regimen was replicated in the 3D-TGA, with and without the stromal component of the assay – human mesenchymal stem cells (hMSC), and with and without the adjunctive PDE5i Vardenafil. Following exposure to drug combinations at human tissue-relevant concentrations, the cell viability was assessed using the alamarBlue® assay (ThermoFisher Scientific, Loughborough, UK). The chemotherapeutic effect of the drugs evaluated in the 3D-TGA is calculated as a percentage of the matched untreated control. Using the Chou Talalay method,^68^ IC_50_ curves are generated for the drugs both individually and in combination as previously described by our group, where drugs in combination were used at constant ratios to make them amenable to synergy testing,^69^ with 10-fold serial dilutions tested starting with the highest final concentrations 200μM Epirubicin/200μM Cisplatin/10000μM 5-Fluorouracil/10μM Vardenafil. Viability curves were generated and IC50 values calculated using GraphPad Prism 5 software (San Diego, CA, USA). The IC_50_ values were compared with the mean peak serum concentrations seen in patients for each of the drugs to evaluate chemotherapeutic response as previously reported^44^ (see table below). The chemotherapeutic response was thus defined as sensitive (IC_50_ below the mean peak serum), borderline (+/- 10% of the mean peak serum), or resistant (IC_50_ above the mean peak serum).

**Table:** Peak serum concentration of chemotherapy agents in humans

Drug	Mean peak serum	Reported publications	Dose in reference
Epirubicin	4.5μM	Cividalli et al.^70^	50mg/m^2^
Cisplatin	4.3μM	Máthé et al. and Johnsson et al.^71^^,^^72^	60 mg/m^2^
Fluorouracil/Capecitabine	4.6μM	Kolinsky et al.^73^	200 mg/m^2^ & 625 mg/m^2^
Vardenafil	0.0267μM	(FDA licensing documentation: https://www.accessdata.fda.gov/drugsatfda_docs/label/2007/021400s010lbl.pdf)	20 mg for ED

Statistical analysis was undertaken to assess the relative efficacy of the drug combinations using a two-way ANOVA to compare the different parameters among the different groups. Difference between groups was only considered to be significant if there was no overlap between the 95% confidence interval about the median. The paired and un-paired t-test was used to calculate the significance of difference between parametrically distributed groups, and Mann-Whitney U test between independent groups, with a significance level of p < 0.05. Statistics were computed with GraphPad Prism 5 software (San Diego, CA, USA) and plotted with mean values, and error bars for standard deviation.

#### IHC readout for CAF differentiation in patient-derived xenografts

IHC for alpha-SMA and periostin was conducted on PDX mice from dose escalation trials of ECX +/- PDE5i. In order to quantify CAF differentiation in PDX tumors, we adapted previously published methods for evaluation of fibrosis in pediatric liver transplants^74^ and stromal reactions in breast cancer.^75^ Whole slides were scanned using a digital slide scanner (Olympus VS110). Quantification of staining was conducted digital whole slide images using QuPath (version 0.2.0).^63^ Automated segmentation of tissue sections was performed on whole slide images followed by thresholding of deconvolved DAB optical density to determine area of tissue stained with anti-alpha-SMA and anti-periostin respectively. A percentage of total tissue area stained by anti-α-SMA or anti-periostin was determined by dividing the DAB thresholded area by the total area of each tissue section and multiplying by 100. Statistical analysis was carried out using R (version 4.1.0, “Camp Pontanezen”). Groovy scripts for measuring alpha-SMA and periostin-expressing tissue in QuPath are available online at Zenodo (https://doi.org/10.5281/zenodo.5222122).

### Quantification and statistical analysis

Statistical analysis was performed with SPSS® version 19 unless otherwise specified (SPSS, Chicago, United States). Kruskal-Wallis, One-way ANOVA, Mann Whitney *U* and T-tests were used to compare groups, as appropriate. *P* < 0.05 was considered statistically significant (P < 0.05∗, P <0.01∗∗, P<0.001∗∗∗, P<0.0001∗∗∗∗). The statistical detail of experiments can be found in the appropriate place in the text, figures and figure legends, where we describe the definition of “n” and given measurements of precision.

## Data Availability

• Standardized datasets:• Whole slide images of mouse PDX models stained with anti-αSMA and anti-periostin antibodies are available to freely download at Zenodo (https://doi.org/10.5281/zenodo.5222122). • The mass spectrometry proteomics data have been deposited to the ProteomeXchange Consortium via the PRIDE partner repository with the dataset identifier PXD031148. • Bulk RNAseq data from 3D-TGAs have been deposited to NCBI GEO with accession number GSE194277. • Custom Computer Code:• Associated Groovy scripts used to analyse the images in QuPath are also available freely on Zenodo (https://doi.org/10.5281/zenodo.5222122). • Any additional information required to reanalyze the data reported in this work paper is available from the Lead Contact upon request.

## Supplemental information

Document S1. Figures S1–S4 and Table S4

Table S1. Log2 ratios for all proteins quantified by mass spectrometry in CAF treated with vardenafil and PDE5A siRNA, and NOF, related to Figure 4

Table S2. Log2 ratios for differentially expressed proteins between CAF and NOF, CAF treated with vardenafil vs untreated and CAF treated with PDE5A siRNA vs control siRNA, related to Figure 4

Table S3. Log2 ratios of differentially expressed proteins that were upregulated in CAF compared to NOF but became downregulated when treated with vardenafil or PDE5A siRNA, related to Figure 4

Document S2. Article plus supplemental information

## References

[1] Alderson D., Cunningham D., Nankivell M., Blazeby J.M., Griffin S.M., Crellin A., Grabsch H.I., Langer R., Pritchard S., Okines A. (2017). Neoadjuvant cisplatin and fluorouracil versus epirubicin, cisplatin, and capecitabine followed by resection in patients with oesophageal adenocarcinoma (UK MRC OE05): an open-label, randomised phase 3 trial. Lancet Oncol..

[2] Allum W.H., Stenning S.P., Bancewicz J., Clark P.I., Langley R.E. (2009). Long-term results of a randomized trial of surgery with or without preoperative chemotherapy in esophageal cancer. J. Clin. Oncol..

[3] Shapiro J., van Lanschot J.J.B., Hulshof M.C.C.M., van Hagen P., van Berge Henegouwen M.I., Wijnhoven B.P.L., van Laarhoven H.W.M., Nieuwenhuijzen G.A.P., Hospers G.A.P., Bonenkamp J.J. (2015). Neoadjuvant chemoradiotherapy plus surgery versus surgery alone for oesophageal or junctional cancer (CROSS): Long-term results of a randomised controlled trial. Lancet Oncol..

[4] Weaver J.M.J., Ross-Innes C.S., Shannon N., Lynch A.G., Forshew T., Barbera M., Murtaza M., Ong C.J., Lao-Sirieix P., Dunning M.J. (2014). Ordering of mutations in preinvasive disease stages of esophageal carcinogenesis. Nat. Genet..

[5] Secrier M., Li X., De Silva N., Eldridge M.D., Contino G., Bornschein J., MacRae S., Grehan N., O'Donovan M., Miremadi A. (2016). Mutational signatures in esophageal adenocarcinoma define etiologically distinct subgroups with therapeutic relevance. Nat. Genet..

[6] Frankell A.M., Jammula S., Li X., Contino G., Killcoyne S., Abbas S., Perner J., Bower L., Devonshire G., Ococks E. (2019). The landscape of selection in 551 esophageal adenocarcinomas defines genomic biomarkers for the clinic. Nat. Genet..

[7] Ross-Innes C.S., Becq J., Warren A., Cheetham R.K., Northen H., O'Donovan M., Malhotra S., di Pietro M., Ivakhno S., He M. (2015). Whole-genome sequencing provides new insights into the clonal architecture of Barrett's esophagus and esophageal adenocarcinoma. Nat. Genet..

[8] Izadi F., Sharpe B.P., Breininger S.P., Secrier M., Gibson J., Walker R.C., Rahman S., Devonshire G., Lloyd M.A., Walters Z.S. (2021). Genomic analysis of response to neoadjuvant chemotherapy in esophageal adenocarcinoma. Cancers (Basel).

[9] Underwood T.J., Hayden A.L., Derouet M., Garcia E., Noble F., White M.J., Thirdborough S., Mead A., Clemons N., Mellone M. (2015). Cancer-associated fibroblasts predict poor outcome and promote periostin-dependent invasion in oesophageal adenocarcinoma. J. Pathol..

[10] Orsulic S., Dahl L., Jensen E., Walts A.E., Andl T., Zhang Y. (2019). Cancer-associated fibroblasts build and secure the tumor microenvironment. Front. Cell Developmental Biol..

[11] Park R., Williamson S., Kasi A., Saeed A. (2018). Immune therapeutics in the treatment of advanced gastric and esophageal cancer. Anticancer Res..

[12] Gorchs L., Fernández Moro C., Bankhead P., Kern K.P., Sadeak I., Meng Q., Rangelova E., Kaipe H. (2019). Human pancreatic carcinoma-associated fibroblasts promote expression of Co-inhibitory markers on CD4+ and CD8+ T-cells. Front Immunol..

[13] Gok Yavuz B., Gunaydin G., Gedik M.E., Kosemehmetoglu K., Karakoc D., Ozgur F., Guc D. (2019). Cancer associated fibroblasts sculpt tumour microenvironment by recruiting monocytes and inducing immunosuppressive PD-1+ TAMs. Sci. Rep..

[14] Nurmik M., Ullmann P., Rodriguez F., Haan S., Letellier E. (2020). In search of definitions: cancer-associated fibroblasts and their markers. Int. J. Cancer.

[15] Desmoulière A., Guyot C., Gabbiani G. (2004). The stroma reaction myofibroblast: a key player in the control of tumor cell behavior. Int. J. Dev. Biol..

[16] Marsh D., Suchak K., Moutasim K.A., Vallath S., Hopper C., Jerjes W., Upile T., Kalavrezos N., Violette S.M., Weinreb P.H. (2011). Stromal features are predictive of disease mortality in oral cancer patients. J. Pathol..

[17] Fink T.L., Francis S.H., Beasley A., Grimes K.A., Corbin J.D. (1999). Expression of an active, monomeric catalytic domain of the cGMP-binding cGMP-specific phosphodiesterase (PDE5). J. Biol. Chem..

[18] Das A., Durrant D., Salloum F.N., Xi L., Kukreja R.C. (2015). PDE5 inhibitors as therapeutics for heart disease, diabetes and cancer. Pharmacol. Ther..

[19] Pantziarka P., Sukhatme V., Crispino S., Bouche G., Meheus L., Sukhatme V.P. (2018). Repurposing drugs in oncology (ReDO)-selective PDE5 inhibitors as anti-cancer agents. ecancermedicalscience.

[20] Aversa A., Bruzziches R., Pili M., Spera G. (2006). Phosphodiesterase 5 inhibitors in the treatment of erectile dysfunction. Curr. Pharm. Des..

[21] Derchi G., Forni G.L. (2005). Therapeutic approaches to pulmonary hypertension in hemoglobinopathies: efficacy and safety of sildenafil in the treatment of severe pulmonary hypertension in patients with hemoglobinopathy. Ann. N. Y Acad. Sci..

[22] Barnes H., Brown Z., Burns A., Williams T. (2019). Phosphodiesterase 5 inhibitors for pulmonary hypertension. Cochrane Database Syst. Rev..

[23] Köhler T.S., McVary K.T. (2009). The relationship between erectile dysfunction and lower urinary tract symptoms and the role of phosphodiesterase type 5 inhibitors. Eur. Urol..

[24] Catalano S., Panza S., Augimeri G., Giordano C., Malivindi R., Gelsomino L., Marsico S., Giordano F., Győrffy B., Bonofiglio D. (2019). Phosphodiesterase 5 (PDE5) is highly expressed in cancer-associated fibroblasts and enhances breast tumor progression. Cancers (Basel).

[25] Zenzmaier C., Kern J., Sampson N., Heitz M., Plas E., Untergasser G., Berger P. (2012). Phosphodiesterase type 5 inhibition reverts prostate fibroblast-to-myofibroblast trans-differentiation. Endocrinology.

[26] Noble F., Lloyd M.A., Turkington R., Griffiths E., O'Donovan M., O'Neill J.R., Mercer S., Parsons S.L., Fitzgerald R.C., Underwood T.J. (2017). Multicentre cohort study to define and validate pathological assessment of response to neoadjuvant therapy in oesophagogastric adenocarcinoma. Br. J. Surg..

[27] Ho W.J., Rooper L., Sagorsky S., Kang H. (2018). A robust response to combination immune checkpoint inhibitor therapy in HPV-related small cell cancer: a case report. J. Immunother. Cancer.

[28] Favi F., Bollschweiler E., Berlth F., Plum P., Hescheler D.A., Alakus H., Semrau R., Celik E., Mönig S.P., Drebber U., Hölscher A.H. (2017). Neoadjuvant chemotherapy or chemoradiation for patients with advanced adenocarcinoma of the oesophagus? A propensity score-matched study. Eur. J. Surg. Oncol..

[29] Gobbini E., Giaj Levra M. (2018). Is there a room for immune checkpoint inhibitors in early stage non-small cell lung cancer?. J. Thorac. Dis..

[30] Wang Q., Ma C., Kemmner W. (2013). Wdr66 is a novel marker for risk stratification and involved in epithelial-mesenchymal transition of esophageal squamous cell carcinoma. BMC Cancer.

[31] Krause L., Nones K., Loffler K.A., Nancarrow D., Oey H., Tang Y.H., Wayte N.J., Patch A.M., Patel K., Brosda S. (2015). Identification of the CIMP-like subtype and aberrant methylation of members of the chromosomal segregation and spindle assembly pathways in esophageal adenocarcinoma. Carcinogenesis.

[32] Kim J., Bowlby R., Mungall A.J., Robertson A.G., Odze R.D., Cherniack A.D. (2017). Integrated genomic characterization of oesophageal carcinoma. Nature.

[33] Ardlie K.G., DeLuca D.S., Segrè A.V., Sullivan T.J., Young T.R., Gelfand E.T., Trowbridge C.A., Maller J.B., Tukiainen T., Lek M. (2015). Human genomics. The Genotype-Tissue Expression (GTEx) pilot analysis: multitissue gene regulation in humans. Science.

[34] Peters C.J., Rees J.R., Hardwick R.H., Hardwick J.S., Vowler S.L., Ong C.A., Zhang C., Save V., O'Donovan M., Rassl D. (2010). A 4-gene signature predicts survival of patients with resected adenocarcinoma of the esophagus, junction, and gastric cardia. Gastroenterology.

[35] Goldman M.J., Craft B., Hastie M., Repečka K., McDade F., Kamath A., Banerjee A., Luo Y., Rogers D., Brooks A.N. (2020). Visualizing and interpreting cancer genomics data via the Xena platform. Nat. Biotechnol..

[36] Garcia E., Hayden A., Birts C., Britton E., Cowie A., Pickard K., Mellone M., Choh C., Derouet M., Duriez P. (2016). Authentication and characterisation of a new oesophageal adenocarcinoma cell line: MFD-1. Sci. Rep..

[37] Sahai E., Astsaturov I., Cukierman E., DeNardo D.G., Egeblad M., Evans R.M., Fearon D., Greten F.R., Hingorani S.R., Hunter T. (2020). A framework for advancing our understanding of cancer-associated fibroblasts. Nat. Rev. Cancer.

[38] Hanley C.J., Mellone M., Ford K., Thirdborough S.M., Mellows T., Frampton S.J., Smith D.M., Harden E., Szyndralewiez C., Bullock M. (2018). Targeting the myofibroblastic cancer-associated fibroblast phenotype through inhibition of NOX4. J. Natl. Cancer Inst..

[39] Saenz De Tejada I., Angulo J., Cuevas P., Fernández A., Moncada I., Allona A., Lledó E., Körschen H.G., Niewöhner U., Haning H. (2001). The phosphodiesterase inhibitory selectivity and the in vitro and in vivo potency of the new PDE5 inhibitor vardenafil. Int. J. Impot Res..

[40] Zenzmaier C., Kern J., Heitz M., Plas E., Zwerschke W., Mattesich M., Sandner P., Berger P. (2015). Activators and stimulators of soluble guanylate cyclase counteract myofibroblast differentiation of prostatic and dermal stromal cells. Exp. Cell Res..

[41] Ilg M.M., Mateus M., Stebbeds W.J., Milenkovic U., Christopher N., Muneer A., Albersen M., Ralph D.J., Cellek S. (2019). Antifibrotic synergy between phosphodiesterase type 5 inhibitors and selective oestrogen receptor modulators in peyronie's disease models. Eur. Urol..

[42] Manousopoulou A., Hayden A., Mellone M., Garay-Baquero D.J., White C.H., Noble F., Lopez M., Thomas G.J., Underwood T.J., Garbis S.D. (2018). Quantitative proteomic profiling of primary cancer-associated fibroblasts in oesophageal adenocarcinoma. Br. J. Cancer.

[43] Macosko E.Z., Basu A., Satija R., Nemesh J., Shekhar K., Goldman M., Tirosh I., Bialas A.R., Kamitaki N., Martersteck E.M. (2015). Highly parallel genome-wide expression profiling of individual cells using nanoliter droplets. Cell.

[44] Saunders J.H., Onion D., Collier P., Dorrington M.S., Argent R.H., Clarke P.A., Reece-Smith A.M., Parsons S.L., Grabowska A.M. (2017). Individual patient oesophageal cancer 3D models for tailored treatment. Oncotarget.

[45] Mishra P.J., Mishra P.J., Glod J.W., Banerjee D. (2009). Mesenchymal stem cells: flip side of the coin. Cancer Res..

[46] Quante M., Tu S.P., Tomita H., Gonda T., Wang S.S., Takashi S., Baik G.H., Shibata W., Diprete B., Betz K.S. (2011). Bone marrow-derived myofibroblasts contribute to the mesenchymal stem cell niche and promote tumor growth. Cancer Cell.

[47] Karnoub A.E., Dash A.B., Vo A.P., Sullivan A., Brooks M.W., Bell G.W., Richardson A.L., Polyak K., Tubo R., Weinberg R.A. (2007). Mesenchymal stem cells within tumour stroma promote breast cancer metastasis. Nature.

[48] Mandard A.M., Dalibard F., Mandard J.C., Marnay J., Henry-Amar M., Petiot J.F., Roussel A., Jacob J.H., Segol P., Samama G. (1994). Pathologic assessment of tumor regression after preoperative chemoradiotherapy of esophageal carcinoma. Clinicopathologic correlations. Cancer.

[49] Hanley C.J., Noble F., Ward M., Bullock M., Drifka C., Mellone M., Manousopoulou A., Johnston H.E., Hayden A., Thirdborough S. (2016). A subset of myofibroblastic cancer-associated fibroblasts regulate collagen fiber elongation, which is prognostic in multiple cancers. Oncotarget.

[50] Bullock M.D., Pickard K.M., Nielsen B.S., Sayan A.E., Jenei V., Mellone M., Mitter R., Primrose J.N., Thomas G.J., Packham G.K. (2013). Pleiotropic actions of miR-21 highlight the critical role of deregulated stromal microRNAs during colorectal cancer progression. Cell Death Dis..

[51] Bhome R., Goh R.W., Bullock M.D., Pillar N., Thirdborough S.M., Mellone M., Mirnezami R., Galea D., Veselkov K., Gu Q. (2017). Exosomal microRNAs derived from colorectal cancer-associated fibroblasts: role in driving cancer progression. Aging (Albany NY).

[52] Ebbing E.A., Van Der Zalm A.P., Steins A., Creemers A., Hermsen S., Rentenaar R., Klein M., Waasdorp C., Hooijer G.K.J., Meijer S.L. (2019). Stromal-derived interleukin 6 drives epithelial-to-mesenchymal transition and therapy resistance in esophageal adenocarcinoma. Proc. Natl. Acad. Sci. USA..

[53] Riedel A., Shorthouse D., Haas L., Hall B.A., Shields J. (2016). Tumor-induced stromal reprogramming drives lymph node transformation. Nat. Immunol..

[54] Chen I.X., Chauhan V.P., Posada J., Ng M.R., Wu M.W., Adstamongkonkul P., Huang P., Lindeman N., Langer R., Jain R.K. (2019). Blocking CXCR4 alleviates desmoplasia, increases T-lymphocyte infiltration, and improves immunotherapy in metastatic breast cancer. Proc. Natl. Acad. Sci. USA..

[55] Costa A., Kieffer Y., Scholer-Dahirel A., Pelon F., Bourachot B., Cardon M., Sirven P., Magagna I., Fuhrmann L., Bernard C. (2018). Fibroblast heterogeneity and immunosuppressive environment in human breast cancer. Cancer Cell.

[56] Lindner T., Loktev A., Giesel F., Kratochwil C., Altmann A., Haberkorn U. (2019). Targeting of activated fibroblasts for imaging and therapy. EJNMMI Radiopharm. Chem..

[57] Thenappan T., Ormiston M.L., Ryan J.J., Archer S.L. (2018). Pulmonary arterial hypertension: pathogenesis and clinical management. BMJ.

[58] Hwang I.C., Kim Y.J., Park J.B., Yoon Y.E., Lee S.P., Kim H.K., Cho G.Y., Sohn D.W. (2017). Pulmonary hemodynamics and effects of phosphodiesterase type 5 inhibition in heart failure: a meta-analysis of randomized trials. BMC Cardiovasc. Disord..

[59] Serafini P., Meckel K., Kelso M., Noonan K., Califano J., Koch W., Dolcetti L., Bronte V., Borrello I. (2006). Phosphodiesterase-5 inhibition augments endogenous antitumor immunity by reducing myeloid-derived suppressor cell function. J. Exp. Med..

[60] Metsalu T., Vilo J. (2015). ClustVis: a web tool for visualizing clustering of multivariate data using Principal Component Analysis and heatmap. Nucleic Acids Res..

[61] Huang da W., Sherman B.T., Lempicki R.A. (2009). Systematic and integrative analysis of large gene lists using DAVID bioinformatics resources. Nat. Protoc..

[62] Satija R., Farrell J.A., Gennert D., Schier A.F., Regev A. (2015). Spatial reconstruction of single-cell gene expression data. Nat. Biotechnol..

[63] Bankhead P., Loughrey M.B., Fernández J.A., Dombrowski Y., McArt D.G., Dunne P.D., McQuaid S., Gray R.T., Murray L.J., Coleman H.G. (2017). QuPath: open source software for digital pathology image analysis. Sci. Rep..

[64] Underwood T.J., Derouet M.F., White M.J., Noble F., Moutasim K.A., Smith E., Drew P.A., Thomas G.J., Primrose J.N., Blaydes J.P. (2010). A comparison of primary oesophageal squamous epithelial cells with HET-1A in organotypic culture. Biol. Cell.

[65] Bergman L.M., Birts C.N., Darley M., Gabrielli B., Blaydes J.P. (2009). CtBPs promote cell survival through the maintenance of mitotic fidelity. Mol. Cell Biol..

[66] Moutasim K.A., Nystrom M.L., Thomas G.J. (2011). Cell migration and invasion assays. Methods Mol. Biol..

[67] Jenei V., Nystrom M.L., Thomas G.J. (2011). Measuring invasion in an organotypic model. Methods Mol. Biol..

[68] Chou T.C. (2010). Drug combination studies and their synergy quantification using the Chou-Talalay method. Cancer Res..

[69] Onion D., Argent R.H., Reece-Smith A.M., Craze M.L., Pineda R.G., Clarke P.A., Ratan H.L., Parsons S.L., Lobo D.N., Duffy J.P. (2016). 3-dimensional patient-derived lung cancer assays reveal resistance to standards-of-care promoted by stromal cells but sensitivity to histone deacetylase inhibitors. Mol. Cancer Ther..

[70] Cividalli A., Cruciani G., Livdi E., Cordelli E., Eletti B., Tirindelli Danesi D. (1998). Greater antitumor efficacy of paclitaxel administered before epirubicin in a mouse mammary carcinoma. J. Cancer Res. Clin. Oncol..

[71] Máthé A., Komka K., Forczig M., Szabó D., Anderlik P., Rozgonyi F. (2006). The effect of different doses of cisplatin on the pharmacokinetic parameters of cefepime in mice. Lab. Anim..

[72] Johnsson A., Olsson C., Nygren O., Nilsson M., Seiving B., Cavallin-Stahl E. (1995). Pharmacokinetics and tissue distribution of cisplatin in nude mice: platinum levels and cisplatin-DNA adducts. Cancer Chemother. Pharmacol..

[73] Kolinsky K., Shen B.Q., Zhang Y.E., Kohles J., Dugan U., Zioncheck T.F., Heimbrook D., Packman K., Higgins B. (2009). In vivo activity of novel capecitabine regimens alone and with bevacizumab and oxaliplatin in colorectal cancer xenograft models. Mol. Cancer Ther..

[74] Varma S., Stéphenne X., Komuta M., Bouzin C., Ambroise J., Smets F., Reding R., Sokal E.M. (2017). The histological quantification of alpha-smooth muscle actin predicts future graft fibrosis in pediatric liver transplant recipients. Pediatr. Transpl..

[75] Catteau X., Simon P., Jondet M., Vanhaeverbeek M., Noël J.C. (2019). Quantification of stromal reaction in breast carcinoma and its correlation with tumor grade and free progression survival. PLoS ONE.

