# Supplementary material for: Phosphodiesterase type 5 inhibitors enhance chemotherapy in preclinical models of esophageal adenocarcinoma by targeting cancer-associated fibroblasts
Source: Cell Rep Med. 2022 Jun 21;3(6):100541. doi: 10.1016/j.xcrm.2022.100541 (PMC9244979; doi:10.1016/j.xcrm.2022.100541)
Supplement: Document S1. Figures S1–S4 and Table S4 [file mmc1.pdf]

Cell Reports Medicine, Volume *3*

Supplemental information

Phosphodiesterase type 5 inhibitors enhance chemotherapy in preclinical models of esophageal adenocarcinoma by targeting cancer-associated fibroblasts

Benjamin P. Sharpe, Annette Hayden, Antigoni Manousopoulou, Andrew Cowie, Robert C. Walker, Jack Harrington, Fereshteh Izadi, Stella P. Breininger, Jane Gibson, Oliver Pickering, Eleanor Jaynes, Ewan Kyle, John H. Saunders, Simon L. Parsons, Alison A. Ritchie, Philip A. Clarke, Pamela Collier, Nigel P. Mongan, David O. Bates, Kiren Yacqub-Usman, Spiros D. Garbis, Zoë Walters, Matthew Rose-Zerilli, Anna M. Grabowska, and Timothy J. Underwood

Supplemental Information

Figure S1

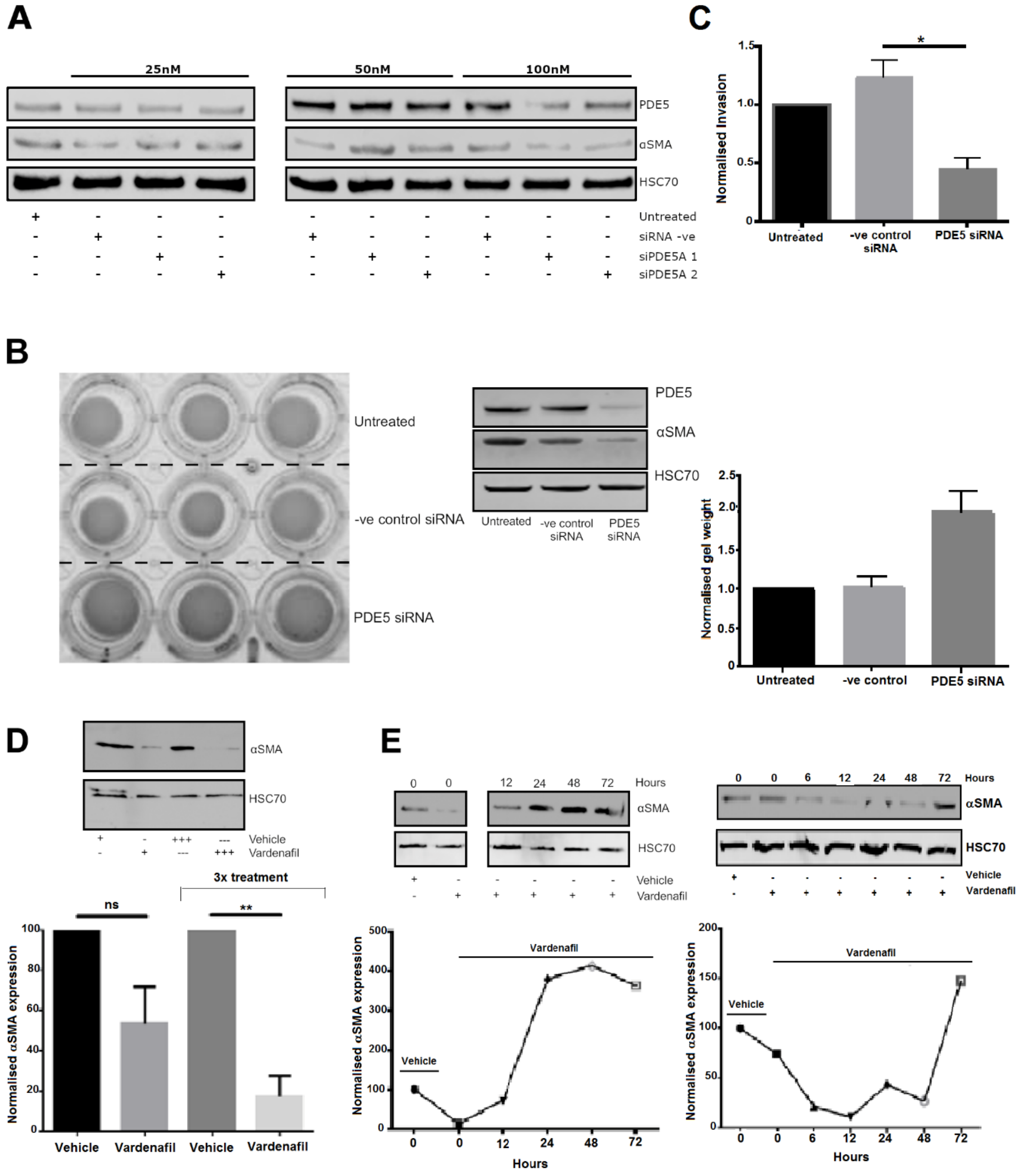

Figure S1. Further assessment of PDE5 inhibition by siRNA and optimisation of PDE5i dosing schedules. Related to Figures 2 and 3. **A.** Western blot showing PDE5 siRNA knockdown in CAFs showing concomitant reduction in PDE5 and αSMA expression. **B.** Fibroblast contraction was assessed using CAFs ± PDE5 siRNA collagen-1 gel contraction assays. Reduction in PDE5 expression reduced collagen-1 gel contraction in CAFs and; **C.** the induction of FLO-1 cell invasion. **D.** Western blot and histogram assessing αSMA expression after 72 hours 50µM vardenafil treatment. A 50 % reduction in expression was achieved with one treatment in 72 hours, whereas treatment every 24 hours (3 treatments in a 72h period) significantly downregulated αSMA expression by 80%. **E.** Western blot assessing αSMA expression in different patient CAFs after the removal of vardenafil treatment. Both CAF populations underwent a significant reduction in αSMA, but comparison of the two CAF populations reveals differences in time to maximum inhibition and time to rebound levels of αSMA expression.

Figure S2

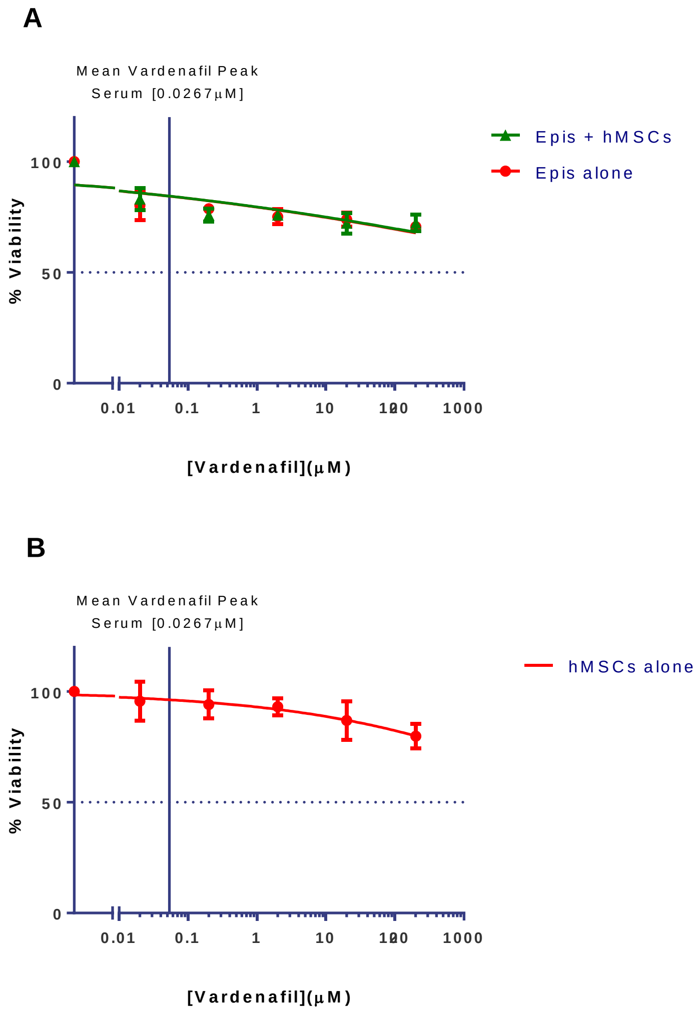

Figure S2. Cells grown in the 3D-TGA do not demonstrate chemo-toxicity upon exposure to the PDE5i Vardenafil at human relevant concentrations. Related to Figure 6. The chemo-sensitivity of esophageal adenocarcinoma (EAC) cancer cell clusters to Vardenafil was determined in 3D-TGA, in 6 replicate wells, after 4 day exposure to the drug at a range of concentrations, using the alamarBlue assay to measure viability. Viability curves were generated and IC50 values calculated using GraphPad Prism. Error bars represent one standard deviation. **(A)** EAC epithelial cells are grown in the 3D-TGA with and without hMSC co-culture before exposure to increasing concentrations of Vardenafil. **(B)** hMSCs are grown without epithelial cancer cells in the 3D-TGA before exposure to increasing concentrations of Vardenafil.

Table S4

Drug	MAGIC regimen	Surface Area to wt ratios conversion	Human dose (at 60kg)	Interspecies conversion factor	Equivalent dose in 20g mouse
**Standard of Care MAGIC regimen:** Repeat cycle every 21 days for 3 cycles pre & post op					
Epirubicin	50 mg/m^2 IV bolus.	/37	1.351mg/kg	x12	16.21 mg/kg
Cisplatin	60 mg/m^2 IV bolus.	/37	1.621 mg/kg	x12	19.46 mg/kg

5-FU	200 mg/m^2 slow IVI	/37	5.40 mg/kg	x12	64.86 mg/kg
Capecitabine (5FU prodrug)	625 mg/ m^2 oral BD	/37	16.89 mg/kg (BD)	x12	202.70 mg/kg (BD)
**PDE5i**					
Vardenafil (short ½ life)	20mg OD		0.333 mg/kg	x12	4 mg/kg
Tadalafil (long ½ life)	40mg OD		0.666 mg/kg	x12	8 mg/kg

Table S4. Derivation of equivalent mouse doses from standard of care regimens for EAC. Related to Figure 7 and STAR methods.

Figure S3

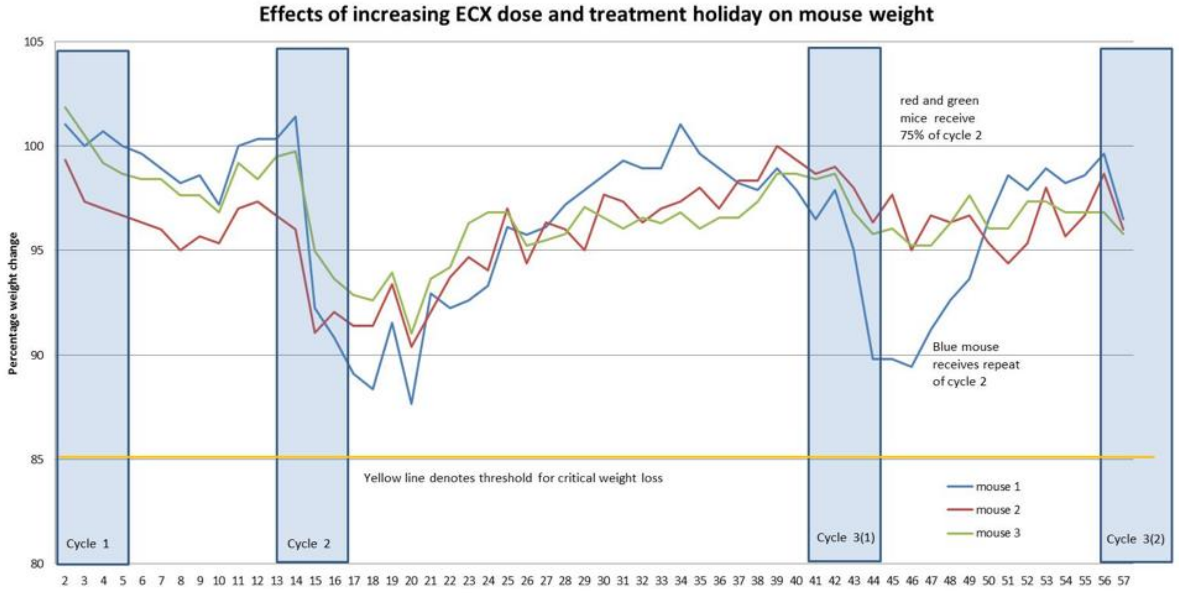

Figure S3. PDE5i dose escalation study in combination with ECX at 50% dosage in non-tumor-bearing mice. Related to Figure 7. Immune-incompetent mice (n=3) tolerate ECX treatment at half the equivalent human dosage. 3 cycles of ECX were administered over 8 weeks. Cycle 1 was 25% equivalent dose; Cycle 2 was 50% equivalent dose; In cycle 3, two mice received 75% equivalent dose and one mouse received a repeat of cycle 2 due to rapid weight loss (50%).

Figure S4

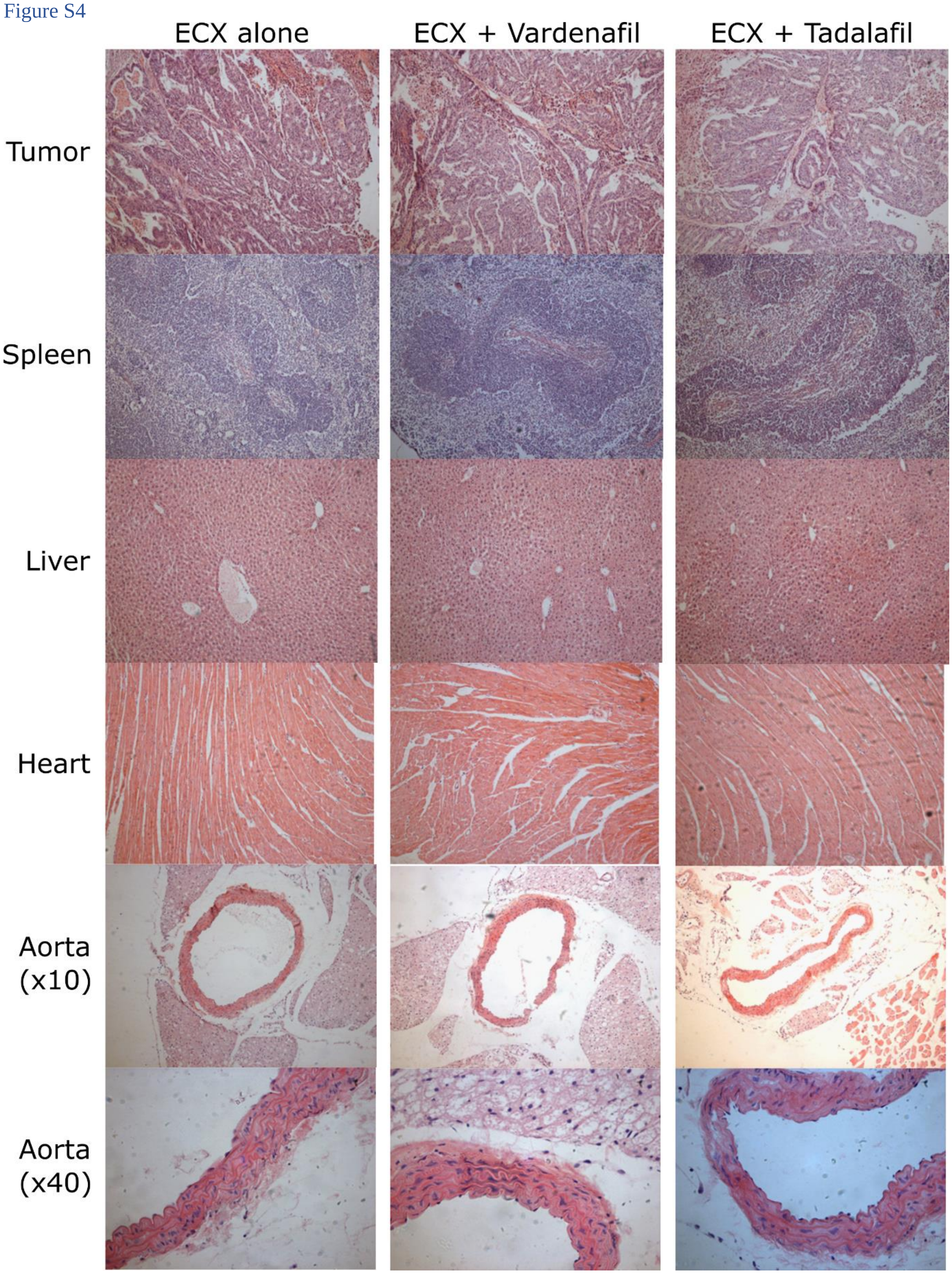

Figure S4. Histological evaluation of PDX tumors and murine organs previously reported as being affected by ECX treatment. Related to Figure 7. Hematoxylin & eosin stained sections of murine tissues previously reported to be histologically affected by administration of high dose PDE5i. PDX-bearing mice were treated with ECX alone or ECX with vardenafil or tadalafil. No gross histological changes were observed in mice treated with vardenafil or tadalafil compared to mice receiving ECX treatment alone.
